# Methodological steps forward in toxicological in vitro screening of mineral wools in primary rat alveolar macrophages and normal rat mesothelial NRM2 cells

**DOI:** 10.1007/s00204-024-03855-7

**Published:** 2024-09-11

**Authors:** Christina Ziemann, Florian Schulz, Christoph Koch, Mette Solvang, Annette Bitsch

**Affiliations:** 1 Fraunhofer Institute for Toxicology and Experimental Medicine ITEM, Nikolai-Fuchs Str. 1, 30625 Hannover, Germany; 2 Technical and Environmental Chemistry, Ernst-Abbe-University of Applied Sciences, Carl-Zeiss-Promenade 2, 07745 Jena, Germany; 3 ROCKWOOL A/S, Group Research and Development, Hovedgaden 584, 2640 Hedehusene, Denmark

**Keywords:** Mineral wools, In vitro, Cytotoxicity, Genotoxicity, Rat alveolar macrophages, Rat mesothelial cells

## Abstract

Man-made vitreous fibers (MMVF) comprise diverse materials for thermal and acoustic insulation, including stone wool. Depending on dimension, durability, and dose, MMVF might induce adverse health effects. Therefore, early predictive in vitro (geno)toxicity screening of new MMVF is highly desired to ensure safety for exposed workers and consumers. Here, we investigated, as a starting point, critical in vitro screening determinants and pitfalls using primary rat alveolar macrophages (AM) and normal rat mesothelial cells (NRM2). A stone wool fiber (RIF56008) served as an exemplary MMVF (fibrous vs. ground to estimate impact of fiber shape) and long amosite (asbestos) as insoluble fiber reference. Materials were comprehensively characterized, and in vivo-relevant in vitro concentrations defined, based on different approaches (low to supposed overload: 0.5, 5 and 50 µg/cm^2^). After 4–48 h of incubation, certain readouts were analyzed and material uptake was investigated by light and fluorescence-coupled darkfield microscopy. DNA-strand break induction was not morphology-dependent and nearly absent in both cell types. However, NRM2 demonstrated material-, morphology- and concentration-dependent membrane damage, CINC-1 release, reduction in cell count, and induction of binucleated cells (asbestos > RIF56008 > RIF56008 ground). In contrast to NRM2, asbestos was nearly inactive in AM, with CINC-1 release solely induced by RIF56008. In conclusion, to define an MMVF-adapted, predictive in vitro (geno)toxicity screening tool, references, endpoints, and concentrations should be carefully chosen, based on in vivo relevance, and sensitivity and specificity of the chosen cell model. Next, further endpoints should be evaluated, ideally with validation by in vivo data regarding their predictivity.

**Supplementary Information:**

The online version contains supplementary material available at 10.1007/s00204-024-03855-7.

## Introduction

One consequence of climate change and the current energy crisis is the increasing demand for energy-efficient-buildings. To achieve this, the choice of insulation products plays an important role. Often, mineral wool products are used, because they combine advantageous properties such as thermal and acoustic insulation as well as non-combustibility. Mineral wool products belong to the group of man-made vitreous fibers (MMVF) that include stone, slag, and glass wools. In North America, glass wools are often referred to as "fiber glass”. Stone wools, for instance, are typically produced from natural rock using a cascade spinning technology. This process yields glassy amorphous fibers with a length-weighted mean diameter of 2.5–4.0 μm and a length of up to 2 mm (Bernstein 2007; Richet 2021). Binders such as phenol-urea–formaldehyde, thermosetting resin, or sugar-based, formaldehyde-free binders (Hjelmgaard et al. 2018) are added during the production process to form the initially loose fibers into a final product. Despite the use of binders, some fibers can become airborne during the manufacturing, installation, and disposal of products. These fibers can pose a risk, when inhaled by humans. Inhalation can occur for fibers if they harbor certain geometric characteristics. Respirable fibers, which can reach the deep lung, are defined as World Health Organization (WHO) fibers exhibiting diameters of < 3 μm, lengths of > 5 μm and a length/diameter ratio of > 3:1 (Andersen et al. 2002). Particularly, respirable fibers longer than 20 µm cannot be engulfed and cleared by macrophages. This can lead to adverse lung effects in humans, such as inflammation and cancer, if the fibers are in addition durable and not cleared by dissolution (Zeider-Erdely et al. 2006). The safety of mineral wool fibers was assessed by the International Agency for Research on Cancer (IARC) of the World Health Organization (WHO) in 2001 with the outcome that mineral wool fibers are not classifiable regarding carcinogenicity to humans (Baan and Grosse 2004). This conclusion was based on in vivo experiments and epidemiological data, considering products as placed on the market, and occupational exposure concentrations. The study also examined the “three Ds” (dose, dimension, and durability) of fiber toxicology (Harrison et al. 2015). While the definition of respirable fiber dimensions has not changed, dose and durability of respirable fibers are still important research topics. Durability represents a key parameter for biopersistence of fibers, however, this endpoint, was not considered by IARC and no differentiation between biopersistent and non-biopersistent fibers is given in its conclusion from 2001 (Andersen et al. 2002). However, certain regulations do make this distinction and both dimension and durability are recognized in legislation, for instance, with Note Q in the European Commission (EC) regulation No 1272/2008 (European Commission 2023) on classification, labelling and packaging of chemicals (CLP). To prove that a mineral wool fiber fulfils the criteria of not being biopersistent, the half-life of respirable fibers is tested in vivo in the rat lung. Using the respective regulatory-relevant method, fibers are considered not to be biopersistent if a half-life of less than 40 days for fibers < 20 µm is shown in a short-term biopersistence test with intratracheal instillation. In turn, durability is largely driven by the chemical composition of a fiber (Hesterberg et al. 2012).

To predict durability and adhere to the 3R principles (Russell and Burch 1959) for animal testing, new fiber types are nowadays often pre-screened in vitro for their bio-solubility using cell-free methods (Barly et al. 2019). Therefore, the dissolution constant of fibers can e.g., be studied in a flow-through cell in artificial lung fluid, as described previously (Okhrimenko et al. 2022). Although such acellular in vitro test systems may help to reduce animal experiments and are less costly and easier to conduct, they are for various reasons (e.g., non-finalized standardization), not yet regulatory implemented to prove non-biopersistence. Nevertheless, there are promising approaches for acellular measurement of dissolution profiles like the “USP apparatus 4” of Hoffman et al. (2023), which might help in standardization. Notably, these in vitro screening methods are exclusively acellular assays.

The limited number of studies conducted so far with MMVF and living, lung-relevant cells (e.g., Ljungman et al. 1994; Luoto et al. 1997; Dörger et al. 2000, 2001; Shinji et al. 2005; Tàtrai et al. 2006; Zeidler-Erdely et al. 2006; Nguea et al. 2008) had not always had the explicit aim of working towards a possible implementation in legislation. However, the inclusion of living cells in both prediction of durability and toxicological in vitro testing may support regulatory acceptance of acellular in vitro results. Therefore, the aim of the present study was to make a step forward in defining cell models, biological endpoints, and methodological determinants, but also pitfalls in in-vitro (geno)toxicity screening of MMVF. This may aid in the prediction of a (geno)toxic and pro-inflammatory potential of new fiber types already during development. Additionally, validated in vitro screening with appropriate endpoints, test conditions, and cell models could support risk assessment and serve as another building block to continuously ensure the safety of workers and consumers.

For the present study, two normal rat-derived cell types, both relevant in the fiber context, i.e., primary rat alveolar macrophages (first site of contact for inhaled fibers in the lung; non-dividing) and normal rat mesothelial NRM2 cells (target cells for asbestos-mediated mesothelioma development; proliferating cells) were chosen. Since regulatory required biopersistence testing is performed in rats, rat cell models were preferred for comparison and relevance reasons. High value was set on fiber characterization, estimation of in vivo-relevant in vitro concentrations, and fiber uptake. Determination of relevant, easy-to-measure endpoints should also be convenient and preferably free of artifacts.

## Materials and methods

### Chemicals and reagents

All used chemicals and solvents were of analytical grade. Most chemicals and salts as well as ethyl methanesulfonate (EMS), Triton^™^ X-100, low-(LMA; peqGold No. 35-2010) and normal-melting point (NMA; peqGold No. 35-1010) agarose, and thioglycolate broth were obtained from Merck/ Sigma-Aldrich Chemie GmbH (Taufkirchen, Germany) and Dulbecco’s Modified Eagle’s cell culture medium (DMEM) with high glucose (4.5 g/L), GlutaMax^™^, sodium pyruvate and gentamicin from GIBCO/Invitrogen (Karlsruhe, Germany). Fetal calf serum (FCS) and normal DMEM were sourced from PAN-Biotech (Aidenbach, Germany) and the ready-to-use solution of 10,000 µg/ml streptomycin sulfate and 10,000 U penicillin G, sodium salt from Euroclone (Pero, Italy). ATCC Kaighn's modification of Ham’s F-12 medium (F-12 K), 24-well plates with hydrophobic culture surface (1.9 cm^2^ cell culture surface per well), one-well Nunc^™^ Lab-Tek^™^ II glass chamber slides, and one-well ClipMax chamber slides were purchased from (Thermo Fisher Scientific, Germany), whereas ethidium bromide solution was obtained from Merck-Millipore (Darmstadt, Germany), Vectashield^®^ H-1000 mounting medium from (BIOZOL, Eching, Germany), the Rat CXCL1/CINC-1 DuoSet^®^ ELISA kit from R&D Systems (Bio-Techne GmbH, Wiesbaden, Germany), the “Cytotoxicity Detection Kit” from Roche Diagnostics (Mannheim, Germany), and purpose-made slides with one roughened surface from Menzel Gläser (Brunswick, Germany).

### Preparation and physicochemical analysis of the exemplary MMVF sample

For the present study, the bio-soluble stone wool fiber RIF56008, formerly shown to be Note Q-compliant in rat lungs after intratracheal instillation, was chosen as exemplary MMVF. RIF56008 was produced by ROCKWOOL A/S (Hedehusene, Denmark). The specific fiber sample was selected from already-sized fiber fractions obtained during in vivo biopersistence testing in compliance with Note Q of Regulation (EC) 1272/2008. According to Note Q, carcinogen classification does not need to apply if the fiber fraction (length > 20 μm) exhibits a weighted half-life of < 40 days. As in vivo biopersistence testing of MMVF is performed with binder-free fibers, binder-free material was also used for the present in vitro screening approach. RIF56008 was originally produced using cascade spinning technology (Richet 2021) and supplied as bulk material (fiber wool). The material density amounted to 2.7 g/cm^3^. Chemical characterization of the bulk material was conducted by the Fraunhofer Institute for Silicate Research ISC (Würzburg, Germany). Chemical analysis of SiO_2_ content was done according to DIN 52340-2:1974. Chemical composition of element oxides was analyzed based on DIN 51086-2:2004, using optical emission spectroscopy inductively coupled to plasma (ICP-OES) and was determined as [wt%]: 38.0 SiO_2_, 18.5 Al_2_O_3_, 0.6 TiO_2_, 8.7 Fe_2_O_3_, 29.2 Σ CaO + MgO, 3.3 Σ Na_2_O + K_2_O plus traces of some other metal oxides. For sizing of RIF56008 bulk material, two-step aerosol separation technique was used. In the first step, the bulk material was aerosolized by a suitable dispersion technique, followed by splitting of the airborne fibers into two fractions using an inertial classifier. The coarse particles and fibers were collected by a virtual impactor, whereas the fine particles and fibers were sampled downstream of the separator using a filter. In the present study, a respirable fiber fraction with a geometric mean diameter (GMD) of 0.81 µm for fibers longer than 20 µm was finally used. The GMD of the WHO fiber fraction amounted to 0.63 µm. Re-characterization of the fiber fraction was conducted within this study to confirm appropriate length and fiber distributions (see below), according to the EU protocol ECB/TM/27 rev.7 (European Commission 1999) and the respective German regulation, i.e., “Technische Regel für Gefahrstoffe” (TRGS) 905 (BAuA 2016), to mimic as far as possible the in vivo testing situation.

### Reference materials

As vehicle controls and exposure media, standard growth media for primary rat alveolar macrophages (AM) and NRM2 cells were used (see “Cell models”). Ground RIF56008 from the same fiber batch (identical chemical composition), also provided by ROCKWOOL A/S, served as non-fibrous, particle-like reference material for differentiation of chemical and morphological effects. The material control was prepared by effectively destroying fiber morphology by grinding, using a Retsch Vibratory Disc Mill RS 200 (Retsch GmbH, Germany) with the disk material made of wolfram carbide. The material was crushed four times for 30 s at a speed of 1200 revolutions per min (rpm) plus six times for 45 s at 1200 rpm. The ground material was characterized afterwards by scanning electron microscopy (SEM) to show effective depletion of fibers and to characterize the obtained particle fraction (for results see Tables 2 and 3). As bio-insoluble fiber reference, long amosite asbestos (Johns Manville Corp., Littleton, CO, USA) was used, which often served as a positive control in fiber carcinogenicity studies. Raw long amosite asbestos was milled for 30 s at full speed, using a Moulinex grinder (Type AR100G31) to obtain respirable material, which was subsequently characterized by SEM to obtain the length and diameter distribution (for results see Tables 2 and 3).

### Analysis of length and diameter distributions and calculation of specific surface area

Length and diameter distributions of RIF56008, RIF56008 ground and amosite asbestos were determined using a scanning electron microscope (SUPRA 55, Carl Zeiss NTS GmbH, Oberkochen, Germany). The general characterization principles used in the context of in vivo biopersistence tests were followed. All materials were initially subjected to low-temperature ashing before being suspended in dispersion medium (Porter et al. 2009; amosite asbestos) or filtered water (RIF56008). Before SEM analysis, the RIF56008 and RIF56008 ground suspensions were sonicated for about 1 min using a Sonorex RK 510H device at 35 kHz and 160 W for 1 min. For amosite asbestos, ultrasonic treatment was done for 10 min using a VS 70 T sonotrode on a Sonoplus HD 2070 ultrasonic homogenizer (Bandelin, Berlin, Germany) at 90% duty cycle and 100% amplitude. Small fractions of the different materials (about 0.01–0.04 mg per 25 mm filter) were then diluted in about 10 ml of filtered water and filtered onto Nuclepore filters (25 mm diameter, pore size 0.2 µm). The fiber containing filter was finally mounted on an aluminum stub and sputtered (Quorum Q 150R ES) with a layer of about 20 nm of gold.

The general guidelines, as described by the World Health Organization's Regional Office for Europe (WHO/EURO) Technical Committee for Monitoring and Evaluating Airborne MMVF (WHO 1985), were followed for counting and size characterization and were adapted to synthetic mineral fibers. In brief, for measurement of length and diameters an SEM magnification of at least 2000× was used. All visible objects were counted. An object was considered as fiber, if the length-to-diameter ratio was at least 3:1. All other objects were considered as particles. Fibers crossing the boundary of the visual field were counted according to the following rules, i.e., fibers with only one end in the field were weighted as half of a fiber, and fibers with neither of their ends in the field were excluded. Fibers diameters were measured at full screen magnification, i.e., up to 18,000×. Length and diameter were recorded individually for each object measured.

**Fig. 1:**
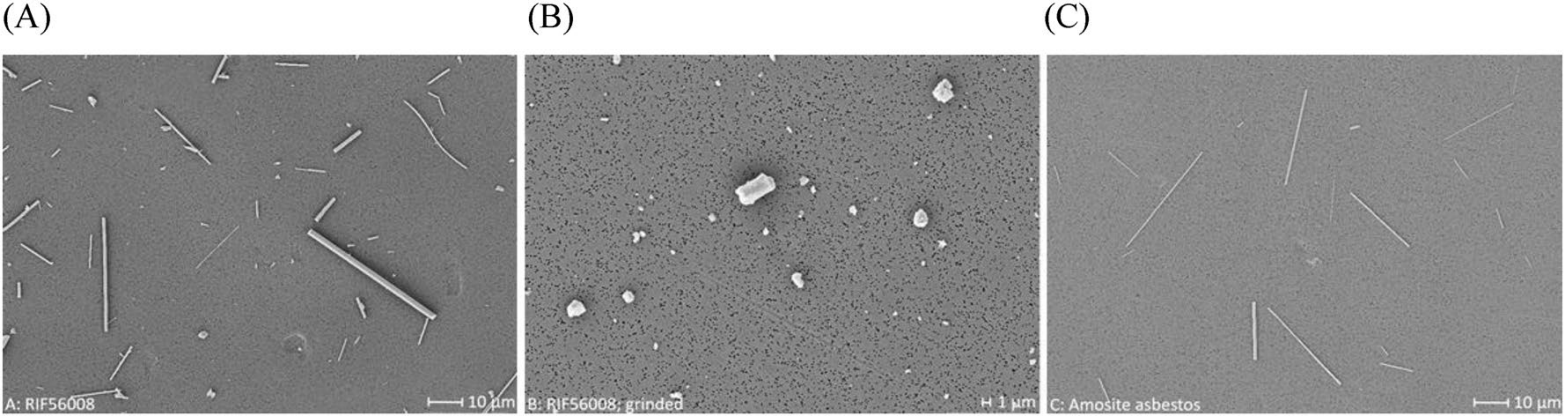
Representative SEM images of the material samples used in the present study. **A** RIF56008 (magnification: 2000×), **B** RIF56008 ground (magnification: 5000×), **C** amosite asbestos (magnification: 2000×)

A total of about 0.15 mm^2^ of the filter surface (for 25 mm filters) was examined. For fibers, a size-selected analysis using a minimum of 100 fibers per category for the two length categories < 5 µm and > 20 µm, and a minimum of 200 fibers for the length category > 5 µm as well as < 20 µm was used. The distance between two visual fields analyzed was at least 10 fields. Sizing was stopped when 1 mm^2^ of the filter surface was examined, even if the minimum number of fibers was not reached for a category. The total number of fibers per filter was determined by normalizing the surface area counted to the total surface area of the filter. For particles, recording was stopped, when a total of 100 particles was reached (for representative SEM pictures see Fig. 1). Additionally, fiber and particle concentrations per mg material were determined for all samples to add in definition of appropriate and relevant concentration levels for cell exposure.

Total specific surface area was finally calculated from the objects measured during SEM analysis, assuming cylindrical geometry for fibers and an ellipsoid shape for particles. Length and diameter values were used to calculate the volume and surface of objects. The mass was determined by multiplying the volume with a standard material density of 2.7 g/cm^3^ for stone wools. To calculate the total surface area the total surface of objects was divided by the total mass of objects.

### Approach for definition of dose metrics and test concentrations

For establishment of a meaningful and predictive MMVF-adapted in vitro screening tool, definition of test concentrations is of utmost importance to avoid artificial results based on overload scenarios. Equivalent human exposure conditions should ideally be mimicked or at least included into dose considerations such as appropriate route of exposure as well as dosimetry aspects. Respirability and deposition fractions of fibers in the respiratory tract regions depend amongst others on shape and size. The used approach for definition of meaningful in vitro concentrations thus focused on respective information gathering, calculations and decision making to obtain a scientifically sound basis for the choice of three relevant in vitro concentrations. Extrapolation from the human external dose to deposition in the lung, and the relationship of lung volume to cell and/or cell culture surface of in the vitro model system was considered. Therefore, different topics are discussed below, i.e., (i) definition of relevant metrics; (ii) concentration definition, referring to human exposure situation at workplaces or in private settings; this implies knowledge of occupational exposure limits or derived threshold values as well as measured exposure data, and (iii) calculation/modelling of deposited dose in the lung.

#### Definition of relevant metrics

While fiber diameter and length influence both deposition, clearance and bioavailability/biopersistence (e.g., Roggli 2015), other dose metrics such as surface area and number of particles are being explored currently as potentially more mechanistically relevant. When comparing different types of particles, the inhaled dose can be expressed in terms of particle volume, particle surface area or number of particles (Oberdörster et al. 1994; Jarabek et al. 2005; Kuempel et al. 2012, 2015). However, in our present approach, we had to compare a mineral wool (RIF56008) and amosite asbestos to a non-fibrous dust control (ground RIF56008). As a common exposure metrics for both fibers and particles was needed, our methodology/approach narrowed down to fiber/particle mass rather than fiber/particle number to be the most adequate metric. Already the used MMVF fraction itself represents a mixture of different size fractions, as does the particle-like material reference. From this point of view, mass is probably the most appropriate and the only possible metric to compare particulate materials of different morphology. Nevertheless, fiber number and specific surface area were reported as additional information and were used for deriving correlations, when considering the two fiber types.

#### Dose definition: thresholds and exposure data

Measured burden of airborne respirable fibers at workplaces is usually low (< 1 fiber/ml air). Recent studies have shown that occupational exposure concentrations have not increased in the last decades (Marchant et al. 2021) and are still below one fiber per cm^3^, which represents the most frequent occupational exposure limit for biopersistent mineral wools. However, exceptions might occur during blowing or spraying operations, i.e., during the insulation of aircraft. Here mean levels of up to 1.8 fibers/ml and 4.2 fibers/ml had been detected for fibrous glass and mineral wools, respectively. Mean concentrations during installation of loose fill in confined spaces have revealed up to 8.2 fibers/ml (Occupational Safety and Health Series No. 64, 1989), whereas values up to 10 mg/m^3^ air were obtained for non-occupational exposure with highest burden assumed in old houses. In Germany, the general dust limit value (“Allgemeiner Staubgrenzwert”) was deduced and laid down in the TRGS 900 (BAuA 2006), which also applies for non-carcinogenic fibers, e.g., mineral wools that had passed the criteria according to Note Q and TRGS 900. This dust limit value was re-evaluated in 2014 by the German Committee on Hazardous Substances (AGS) and was set to 1.25 mg/m^3^ respirable dust (“Alveolengängige Fraktion”, “A-Staub”; referred to a density of 2.5 g/cm^3^). The Permanent Senate Commission for the Investigation of Health Hazards of Chemical Compounds in the Work Area of the “Deutsche Forschungsgemeinschaft” (MAK Commission) has defined a limit value for granular biopersistent dust in a comparable range (0.3 mg/m^3^ respirable dust, referring to a density of 1 g/cm^3^) (DFG 2014).

#### Deposited dose in the lung as a basis for in vitro testing

To define a meaningful, data-derived in vitro concentration, conversion of an external exposure level or limit value to a resulting internal deposited dose on the lung surface is necessary. In a first step, the concentration for the RIF56008 fiber sample was defined, and the concentrations of the material reference i.e., ground RIF56008 was then adapted to the fiber sample concentration.

Initially, the GMD of the WHO fiber fraction, as obtained from SEM measurements was subjected to multiple path particle dosimetry (MPPD) calculations. The MPPD model provides a mechanistic modeling to predict deposition and retained doses in lung and has been used in various applications to predict doses of inhaled particles, including elongated mineral particles (e.g., Jarabek et al. 2005; NIOSH 2013; Asgharian et al. 2018). For respective calculations, default values and MPPD settings (human, MPPD 3.04), as defined in TRGS 910 (BAuA 2014) were used (see Supplementary Tab. S1). In addition, models for both rat and human were calculated to bridge the results from in vivo biopersistence studies to the hazard for human beings at workplaces or in private settings. However, MPPD was originally designed for particles and mass median aerodynamic diameter (MMAD) values are a prerequisite for calculation. The MAK Commission stated that for particles including fibrous structures with a diameter of > 0.5 μm the aerodynamic diameter is always the most relevant dimension. Here, the aerodynamic diameter is essentially determined by the diameter with length being of lower influence. For long fibers (length >  > diameter) the MAK Commission concluded on an aerodynamic diameter of 3 times the fiber diameter, supported by data from Sturm et al*.* (2009, 2021), who theoretically modelled the deposition and clearance of fibers with variable sizes. The authors had chosen for their mathematical modelling of deposition and clearance, fibers with an aspect ratio varying between 3 and 100 and a diameter ranging from 0.001 to 10 µm to cover a broad spectrum of inhalable particles.

As an approach, the general dust limit value (1.35 mg/m^3^ for a density of 2.7 g/cm^3^) served as an appropriate external exposure concentration, and the GMD values obtained by SEM (see Table 2), multiplied by a factor of three were used as MMAD values. A density of 2.7 g/cm^3^ was assumed to be applicable for mineral wools and 3.4 g/cm^3^ was used for amosite asbestos. Respective results for the deposited fractions are depicted in Table 1 and served as a basis for calculation of the deposited mass per cm^2^ alveolar surface.

**Table 1:** Calculation of theoretical deposition fractions in human lung using MPPD 3.04

	RIF56008	Amosite asbestos
Deposition fraction in conducting airways	0.0344	0.0394
Deposition fraction in alveolar region	0.1031	0.1012
Airway deposition fraction	0.1375	0.1407
Deposited mass/cm^2^ alveolar surface (per min) [µg/cm^2^]	4.62 × 10^−6^	4.53 × 10^−6^
Deposited mass/cm^2^ alveolar surface (working day) [µg/cm^2^]	0.00222	0.00218
Deposited mass/cm^2^ alveolar surface (working year) [µg/cm^2^]	0.53	0.52

The present aim was to make a step forward in developing a predictive in vitro screening tool, adapted to MMVF, ideally using human-relevant doses, and taking into account the difference between short-term tests and long-term exposures in humans. But sensitivity of the in vitro test systems used must also be considered adequately. For this reason, we had a closer look into results from in vitro studies carried out with amosite asbestos. In primary human mesothelial LP9 cells, amosite asbestos showed a small but significant effect on cytotoxicity after 24 h of incubation at a concentration of 5 µg/cm^2^ cell culture surface. Cell proliferation was inhibited, but lactate dehydrogenase (LDH) release, indicative for membrane damage, was not increased (Reamon-Büttner et al. 2021). In another study, focusing on formation of reactive oxygen species (ROS), asbestos showed effects in the range between 2.5 and 10 µg/cm^2^ (Hansen and Mossman 1987). Ljungman et al. (1994) detected an asbestos-induced tumor necrosis factor-alpha (TNF-α) release at about 20 µg/cm^2^. From our considerations about dose (metrics) and the calculations of a human equivalent dose (deposited mass on alveolar surface is in the range of 0.5 µg/cm^2^ per working year) in combination with results from in vitro testing of asbestos fibers (effects at ≥ 5 µg/cm^2^), we finally suggested 0.5 (in vivo-relevant concentration for occupational exposure), 5 (in vitro concentration at which initial effects are expected to occur) and 50 µg/cm^2^ (supposed overload concentration) to represent meaningful in vitro concentrations.

### Cell models

#### Primary rat alveolar macrophages

For this orienting study, primary rat alveolar macrophages (AM) were used as one of the two lung-relevant in vitro cell models. AM are the first side of contact for fibers in the lung and represent a very sensitive test system for in vitro screening experiments with particulate matter (Ziemann et al. 2014, 2017). Cells were isolated from healthy Wistar rats [strain Crl:WI(Han); Charles River, Sulzfeld, Germany] by bronchoalveolar lavage in compliance with the Federal Act on the Protection of Animals (“Tierschutzgesetz”, Bonn, Germany, last revised December 20, 2022). After centrifugation of the cell containing lavage fluid (300 × g, 10 min, 4 °C), the supernatant was discarded, the cell pellet resuspended in cell culture medium, and cells counted and plated at a density of 1.2 × 10^5^ cells in 500 µl of cell culture medium in 24-well plates with hydrophobic culture surface (1.9 cm^2^). To estimate material uptake by fluorescence-coupled darkfield microscopy, AM were plated at a density of 6 × 10^5^ cells in 2 ml cell culture medium in one-well Nunc^™^ Lab-Tek^™^ II glass chamber slides. Before being exposed to the test and reference materials, AM were pre-cultured for 24 h in DMEM with high glucose (4.5 g/l), GlutaMax^™^, and sodium pyruvate (110 mg/l), supplemented with 10% FCS and 5 ml of a ready-to-use solution of 10,000 µg/ml streptomycin sulfate and 10,000 U penicillin G, sodium salt per 500 ml cell culture medium and at 37 °C and 5% CO_2_ in a humidified atmosphere using an incubator.

#### Normal rat mesothelial cells

Normal rat mesothelial (NRM2) cells, as target cells for asbestos-mediated mesothelioma development, served as second cell model. NRM2 cells were a gift of Jeffrey Everitt, MDV, Animal Pathology Core, Duke University School of Medicine (Durham, NC, USA) through James C. Bonner, Department of Biological Sciences, North Carolina State University (Raleigh, NC, USA). For characterization of this normal rat mesothelial cell line see Rutten et al. (1995). Cells were cultured in a 1:1 mixture of ATCC F-12 K and normal DMEM medium, supplemented with 10% FCS-standard and 0.01% gentamicin. Cells were passaged twice a week. For experiments, 5 × 10^4^ cells were plated in 24-well plates and pre-cultured for 24 h, before treatment with the particulate materials. To estimate material uptake by fluorescence-coupled darkfield microscopy, NRM2 cells were plated at a density of 5 × 10^5^ cells in 2 ml cell culture medium in one-well ClipMax chamber slides, and for counting of binucleated cells or mitotic phases, 2.5 × 10^5^, NRM2 cells were plated in one-well ClipMax chamber slides in 3 ml of cell culture medium and were again pre-cultured for 24 h before treatment start.

### Sterility testing and treatment of cells

RIF56008, ground RIF56008, and amosite asbestos were initially tested for sterility by adding a defined amount of the fiber/particle suspensions to thioglycolate broth and incubating two independent samples per material at 34/35 °C for 14 days. Saline (0.9%) served as negative and *Bacillus subtilis* (DSM 10, DSMZ-German Collection for Microorganisms and Cell Cultures, Brunswig, Germany) as positive control. After 14 days, turbidity was checked by the naked eye. Additionally, endotoxin was measured by a commercial service laboratory (Lonza Verviers SPRL, Verviers, Belgium), as endotoxin might lead to unspecific pro-inflammatory effects, and might disturb Enzyme-linked Immunosorbent Assay (ELISA) measurement of the cytokine-induced neutrophil chemoattractant 1 (CINC-1). Sterility and endotoxin testing both did not point to relevant contaminations with bacteria or fungi. Endotoxin was not detected, even at the lowest dilution (1:10). All values were below the detection limit of 0.05 EU/ml.

For cell treatment, the different materials were accurately weighed, sterilized for 4 h at 160 °C in a drying oven and then dispersed in the respective cell culture medium to generate concentrated stock dispersions. Stock dispersions were subsequently homogenized by ultrasonication for 1 min (RIF56008 and ground RIF56008) using a Sonorex Super RK 514 BH ultrasonic water bath or for two times 5 min using a VS 70 T sonotrode on a Sonoplus HD 2070 ultrasonic homogenizer (both Bandelin, Berlin, Germany) at 90% duty cycle and 100% amplitude (amosite asbestos). After sonication the resulting stock dispersions were finally diluted with cell culture medium to get two-fold (AM) or finally concentrated (NRM2) incubation dispersions. The experiments were performed with the carefully chosen concentrations of 0.5, 5, and 50 µg/cm^2^, corresponding to 1, 10, and 100 µg/ml incubation volume.

For AM, 500 µl of the twofold-concentrated incubation dispersions for both the alkaline comet and LDH release assays (performed in parallel), cell counting, and CINC-1 release were then carefully added to the respective cell-containing wells of 24-well plates, resulting in a total incubation volume of 1 ml/well. For fluorescence-coupled darkfield microscopy, 2 ml of the twofold-concentrated incubation dispersions were added to cell-containing one-well glass chamber slides. As negative/vehicle control, 500 µl (24-well plate) or 2 ml (glass chamber slides) of cell culture medium were added to the respective culture vessels. For NRM2 cells, a complete medium exchange was performed and 1 ml (24-well plates) or 4 ml (one-well chamber slides) of the finally concentrated material dispersions or vehicles were added. Both cell types were then exposed to the different fibers/particles or the vehicle controls for 4, 24 or 48 h (depending on the endpoint) at 37 °C and 5% CO_2_ in a humidified culture atmosphere using an incubator. The methodological positive control cultures received 500 µl (AM) or 1 ml (NRM2) of cell culture medium during the incubation period, and the methodological positive controls ethyl methanesulfonate (EMS) and Triton^™^ X-100 were added to the respective wells for 1 h or 5–15 min, respectively, before the end of cell incubation.

At the end of cell treatment, samples of the culture medium were carefully taken for analysis of LDH activity and CINC-1 release in the respective experiments. For determination of DNA strand breaks using the comet assay, and for automatic cell counting, AM were placed on ice for 10 min to enable cell detachment without usage of enzymes like trypsin, to avoid unspecific membrane damage or cell activation. For cell detachment, NRM2 cells were trypsinized and then subjected to the comet assay procedure or automatic cell counting. Determination of cell number, as one endpoint for fiber screening, was done for both cell types using an automatic CASY cell counting device (OLS, OMNI Life Sciences, Bremen, Germany), CASYton isotonic measuring buffer, and CASYcups as measuring vessels, and setting cell-type specific size borders.

### Estimation of cellular uptake

As an aid in interpretation of cyto- and genotoxicity data, uptake of the different particulate materials was estimated using fluorescence-coupled darkfield microscopy. Therefore, AM and NRM2 cells grown and incubated for 24 h in glass chamber slides/one-well ClipMax chamber slides, were washed, subsequently fixed with cold methanol/acetic acid solution (3:1), air dried, and finally stained with 4′,6-diamidino-2-phenylindole (DAPI). Slides were then mounted using Vectashield^®^ H-1000 and particle/fiber internalization was visualized and documented using an enhanced dark field illumination system in fluorescence mode (CytoViva^®^, Auburn, AL, USA), attached to a standard light microscope. Additionally, light microscopy served as a screening tool for evaluation of both cell density, cellular uptake, and cell morphology as well as for estimation of density and homogeneity of the fiber/particle dispersions. Light-microscopic pictures were taken using a camera-equipped Nikon ECLIPSE TS 100 infinity-corrected inverse microscope.

### Lactate dehydrogenase (LDH) release assay

LDH release, indicative for membrane damage, was chosen as cytotoxicity endpoint, as it had previously been used to compare different MMVF in vitro in rat alveolar macrophages (e.g., Luoto et al. 1994), and had also been shown to respond to fiber treatment in vivo, as measured in cell-free bronchoalveolar lavage fluid (e.g., Adamis et al. 2001). To measure LDH release, culture supernatants were sampled at the end of cell treatment, centrifuged at 425 × g for 10 min to clean the supernatants from residual fibers/particles and stored in 1.5 ml reaction tubes. LDH activity was subsequently measured by transferring 100 µl of supernatant per well into a 96-well plate and adding 100 µl of reaction mixture of the “Cytotoxicity Detection Kit”. After incubation for about 15 min at room temperature in the dark, photometric measurement at 490 and 630 nm was performed, using a microplate reader. Percent cytotoxicity was finally calculated using the delta optical density (OD) of the two wavelengths, subtracting the blank value, and setting the negative/vehicle control values to 1 (main tests) or the result of the Triton^™^ X-100-treated cells to 100% (mechanical influence on membrane damage) to finally calculate relative cytotoxicity.

### In vitro alkaline comet assay

To look for DNA strand break induction, cells were subjected to the in vitro alkaline comet assay according to Singh et al. (1988) after 24 h of pre-culture followed by 24 h of incubation. The comet assay represents an indicator test for detection of genotoxicity, which was previously used with AM to estimate the genotoxic potential of alkaline earth silica wools (Ziemann et al. 2014).

In the in vitro alkaline comet assay, all steps after the end of fiber/particle treatment were done under red light to avoid unspecific DNA damage due to UV‐irradiation. Detached cells were transferred to 1.5 ml reaction cups and pelleted by centrifugation for 5 min (900 rpm; Heraeus Biofuge 15, Thermo Scientific, Germany). Cells were subsequently re‐suspended in 80 μL of 0.75% (w/v) pre‐conditioned LMA and applied to purpose-made slides with one roughened surface, which had been pre‐coated with 0.5% (w/v) of NMA. The gels were then overlaid with a cover slip, allowed to set at 4 °C, before adding an additional layer of 100 μL of 0.75% LMA, which was again covered. Slides were then transferred to 4 °C. After removal of the cover slips, the slides were incubated in lysis solution (2.5 M NaCl, 100 mM Na_2_EDTA, 10 mM Tris‐HCl, 8 g/L NaOH, 1% Triton^™^ X-100, 10% dimethyl sulfoxide) overnight at 4 °C. After cell lysis, slides were placed into a pre‐cooled horizontal electrophoresis tank (Agagel Maxi, Biometra, Germany) and covered with pre‐cooled electrophoresis buffer (300 mM NaOH, 1 mM Na_2_EDTA, pH > 13). DNA was allowed to unwind for 20 min to generate DNA-single strand breaks, before electrophoresis was performed at fixed 0.7 V/cm and 300 mA for 20 min. Finally, slides were removed, neutralized by three changes of neutralizing buffer (0.4 M Tris‐HCl pH 7.4), and stained with 80 μL of a 20 μg/mL ethidium bromide solution.

Coded slides were subsequently analyzed microscopically for induction of DNA damage using a camera-equipped Axioskop fluorescence microscope (Carl Zeiss, Göttingen, Germany) with a 40x/0.9 mm Korr Plan-Neofluar Ph3 objective and the Comet Assay III software (Perceptive Instruments, Bury St Edmunds, UK). As a measure for DNA damage, DNA migration out of the cell nucleus was analyzed with the amount of DNA in the comet tail, i.e., the tail intensity (TI) as main and most accepted measure for DNA migration, as given in OECD 489 (OECD 2016). Slides were analyzed under the following criteria i.e., acceptable staining, evaluation of at least 100 nuclei per slide, evaluation of nuclei in the middle of the slide only, avoiding regions with bubbles, and no analysis of overlapping nuclei/comets. Comets without head, also called “hedgehogs” were excluded from analysis. Finally, the median of the single cell data per slide, the mean TI of three replicates per experiment and the means ± SD of the mean TI of three independent experiments were calculated.

### CINC-1 release

To look for release of CINC-1, as a pro-inflammatory chemokine marker, the incubation supernatants of AM and NRM2 cells were frozen and stored at − 80 °C until measurement. CINC-1 was quantified (undiluted for AM, diluted 1:10 for NRM2) using a CINC-specific ELISA kit in 96-well plates (i.e., Rat CXCL1/CINC-1 DuoSet ELISA). Measurements were performed according to the manufacturer’s protocol. OD of each well was finally measured at 450 nm. Wavelength correction was performed automatically using a wavelength of 570 nm, which was subtracted from the 450 nm readings to correct for optical imperfections in the plate.

### Counting of binucleated NRM2 cells

As certain asbestos and glass and stone wool fibers were previously shown to induce bi- and multinucleated cells in human mesothelial cells (Pelin et al. 1995), analysis of binucleated NRM2 cells was included as fiber morphology-dependent endpoint, most likely based on disturbance of cell division by physical effects on both chromosomes and cytoskeleton. For counting of bi-/multinucleated cells, NRM2 cells, pre-cultured in one-well ClipMax chamber slides, were exposed to RIF56008, ground RIF56008 or amosite asbestos for 48 h to enable sufficient cell division. Cultures were then washed, subsequently fixed with cold methanol/acetic acid solution (3:1), air dried, and stained using a standard Giemsa staining protocol. Two thousand NRM2 cells were then analyzed light microscopically for occurrence of bi-/multinucleated cells using a Leica DM4000 B automated upright light microscopy system equipped with a Leica N PLAN L 100x/0.75 objective and a DFC295 camera. As a measure for cytotoxicity/cell proliferation, automatic cell counting was performed using parallel cultures.

### Statistical analyses

For LDH release arithmetic means of at least three independent experiments with three biological replicates each and for CINC-1 release, arithmetic means of up to five biological replicates, measured in duplicate, were subjected to statistical analysis using the Student’s *t*‐test for unpaired values, two-tailed, combined with normality (Shapiro–Wilk) and equal variance testing (Brown–Forsythe). For the in vitro alkaline comet assay mean TI values of the three independent experiments (three biological replicates each) were statistically analyzed. Samples were calculated from the median TI values of the three biological replicates derived from at least 100 nuclei per replicate. Due to the hierarchical nature of in vitro comet assay data and concentration-dependent change of single cell data variance (Møller and Loft 2014), equal variance was not assumed. Therefore, comet assay data were statistically analyzed using the Welch’s *t*-test, one-tailed, combined with normality (Shapiro–Wilk) and equal variance testing (Brown-Forsythe) for pairwise comparison to the negative control. Based on the right skewed data distribution, log transformation of comet assay data might be an option for statistical evaluation but can render testing hypersensitive in combination with the Welch’s *t*-test. The appropriate statistical method for statistical evaluation of in vitro comet assay data is still under debate. This also includes pairwise versus multiple testing approaches. Differences from the negative control were considered statistically significant at *p* ≤ 0.05. “Pearson Product Moment Correlation” was used for detection of correlations between material characteristics and biological effects. All statistical tests were done using SigmaPlot 14.0 (Systat Software GmbH, Germany).

## Results

### SEM analysis of the fiber and particle samples

To characterize both morphology (see Fig. 1 for representative images) and length and diameter distribution of the three investigated materials (Table 2) SEM analyses were performed. Concentrations of the fractions “Fibers”, “Fibers shorter than 5 µm”, “Fibers longer than 5 µm (WHO fibers)”, “Fibers longer than 20 µm” and “Fibers with a length between 5 and 20 µm” as well as “Particles” were subsequently calculated (Table 3).

**Table 2:** Fiber and particle size distributions of the investigated material samples

	RIF56008	RIF56008 ground	Amosite asbestos
Geometric mean length ± SD [µm]			
Total fibers*	5.98 ± 2.38	n.a	6.74 ± 2.64
WHO fibers**	11.60 ± 1.90	n.a	12.63 ± 1.98
Fibers > 20 µm***	32.43 ± 1.52	n.a	32.94 ± 1.54
Particles	1.75 ± 1.54	0.78 ± 1.70	1.66 ± 1.45
Geometric mean diameter ± SD [µm]			
Total fibers*	0.47 ± 1.69	n.a	0.31 ± 1.79
WHO fibers**	0.57 ± 1.66	n.a	0.37 ± 1.71
Fibers > 20 µm***	0.78 ± 1.62	n.a	0.48 ± 1.63
Particles	1.16 ± 1.54	0.61 ± 1.69	0.83 ± 1.43

*n.a.* Not applicable, due to complete conversion to non-fibrous particles

*Fiber definition: length-to-diameter ratio > 3:1

**Fibers with length > 5 µm and diameter < 3 µm

***Fibers with length > 20 µm and diameter < 3 µm 1, relevant fiber fraction according to Note Q of Regulation (EC) 1272/2008

**Table 3:** Number concentrations of fibers and particles in the investigated material samples

Concentration [10^6^/mg]	RIF56008	RIF56008 ground	Amosite asbestos
Total fibers	68.4	0	257.3
WHO fibers	35.6	0	154.8
Fibers < 5 µm	32.9	0	102.5
Fibers > 20 µm	7.2	0	39.1
Particles	19.1	409.6	2.7

Re-characterization of the respirable RIF56008 stone wool fraction (Fig. 1A), indicated a GMD of 0.78 µm for the > 20 µm fraction and of 0.57 µm for the WHO fiber fraction (Table 2). For biopersistence studies according to the EU protocol ECB/TM/27 rev.7, the measured GMD of the long fiber fraction should be as close as possible to 0.8 µm, which was fulfilled for the tested material. Thus, RIF56008 was considered to represent an appropriate model fiber for the present study. Furthermore, the geometric mean length (GML) values of RIF56008 (fibers > 20 µm: 32.43 µm; WHO fibers: 11.60 µm) were nearly identical to the GML values measured for amosite asbestos (fibers > 20 µm: 32.94 µm; WHO fibers 12.63 µm).

To distinguish between material- and fiber-specific effects, ground RIF56008 was included as particle-like material control (Fig. 1B) with the same chemical composition as the exemplary model fiber RIF56008. SEM characterization confirmed an effective grinding process, as no fibers at all were detected and GMD and GML amounted to 0.61 and 0.78 µm, respectively (Tables 2 and 3).

The insoluble fiber reference amosite asbestos (Fig. 1C) was already used as fiber positive control in preceding experiments at Fraunhofer ITEM. Re-characterization of the milled long amosite asbestos by SEM indicated GMDs of 0.48 µm for the fibers > 20 µm and 0.37 µm for the WHO fibers (Table 2). GMLs amounted to 32.94 and 12.36 µm, respectively. For the WHO fiber fraction data were comparable to the respective values given by Rittinghausen et al. (2014), i.e., 0.39 (GMD) and 13.95 µm (GML). Due to high similarity in dimensions, the used sample was supposed to be as active as in the former study and to represent an insoluble fiber positive control.

Notably, RIF56008 and amosite asbestos clearly differed in fiber and particle number concentrations per mg test material. While concentrations of fibers > 20 µm and WHO fibers for RIF56008 were calculated to be 7 × 10^6^ and 35 × 10^6^ per mg test material, respectively, the corresponding values were about 4.3- and 5.6-fold higher for amosite asbestos (Table 3). In contrast, the number of particles was considerably, about sevenfold, lower in the amosite asbestos sample, compared to the RIF56008 fiber sample.

Furthermore, the total specific surface area was calculated from the objects measured by SEM. With 2.21 m^2^/g, long amosite asbestos exhibited the highest specific surface area, while the lowest value was found for ground RIF56008 as non-fibrous material control (1.09 m^2^/g). For RIF56008 a specific surface area of 1.54 m^2^/g was estimated.

#### Cellular uptake of materials

**Fig. 2:**
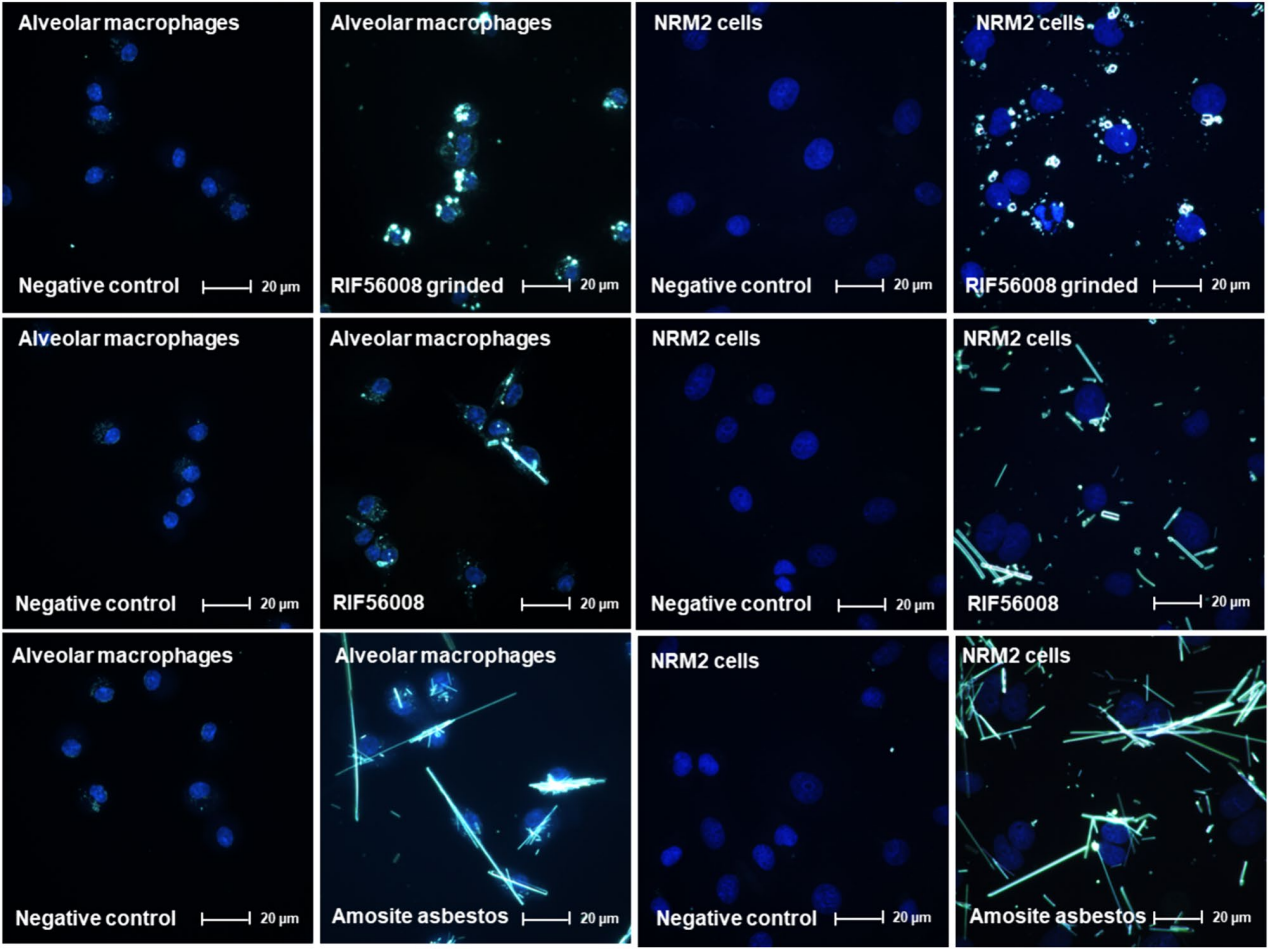
Representative fluorescence-coupled darkfield microscopy pictures from cellular uptake experiments with rat alveolar macrophages and NRM2 cells. Rat alveolar macrophages or NRM2 cells were incubated for 24 h without (negative control) or with 5 µg/cm^2^ of RIF56008 ground, RIF56008, or amosite asbestos (light structures). Cells were subsequently fixed and cell nuclei stained with DAPI (blue round structures). Fluorescence-coupled darkfield microscopy was performed using a 100× oil objective and a final magnification of 1000× (color figure online)

Internalization of RIF56008 ground, RIF56008 and amosite asbestos into AM and NRM2 cells was investigated by fluorescence-coupled darkfield microscopy (Fig. 2). The used incubation time of 24 h represented an incubation time, which should clearly enable material uptake at least into macrophages. Material uptake was clearly concentration-dependent, but for clarity reasons representative pictures are given for 5 µg/cm^2^ only.

After 24 h both AM and NRM2 cells were shown to internalize RIF56008 ground, RIF56008 and amosite asbestos, as concluded from DAPI-stained cell nuclei and the materials being in the same focus plane. The materials clearly accumulated around the cell nuclei. At supposed fiber overload (50 µg/cm^2^), it was nearly impossible to estimate material uptake (see Supplementary Fig. S1), particularly for amosite asbestos, which exhibited considerably higher total and WHO fiber numbers per mass than RIF56008 (Table 3). AM incubated with ground RIF56008 were packed with particles and particle agglomerates. For RIF56008, both particle- and fiber-like structures were visible within the cells. Amosite asbestos demonstrated the highest amount of internalized material, consisting of shorter and longer fiber-like structures. In AM different cells dealt with the same long fiber (Fig. 2 and Supplementary Fig. S2), an already known phenomenon. In NRM2 cells, comparable observations were made, with even higher uptake of the fiber materials (Fig. 2). At 50 µg/cm^2^, some NRM2 cells were nearly fully packed with fibers (Supplementary Fig. S1).

#### Lactate dehydrogenase (LDH) release

**Fig. 3:**
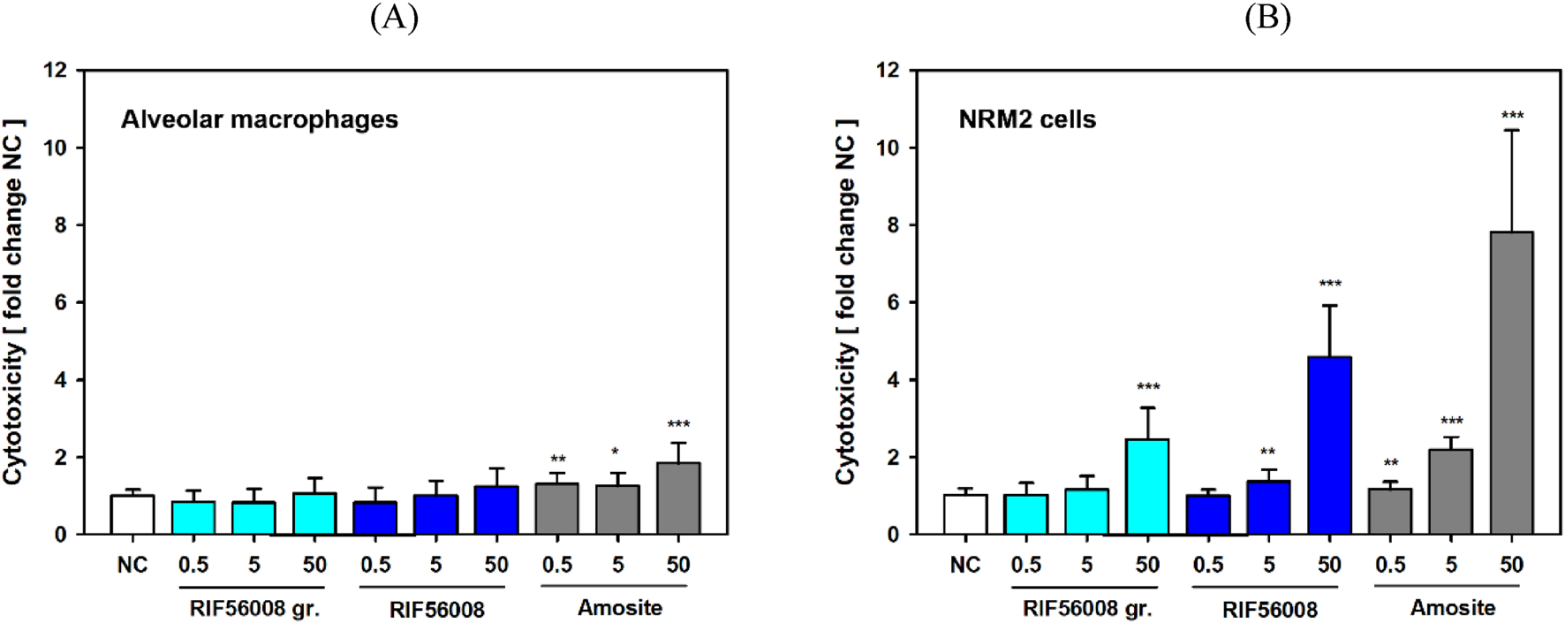
LDH release from rat alveolar macrophages or NRM2 cells after 24 h of incubation with the particulate materials. Rat alveolar macrophages (**A**) or NRM2 cells (**B**) were incubated for 24 h with the given concentrations of RIF56008 or the reference items RIF56008 ground (RIF56008 gr.) or amosite asbestos (Amosite), before sampling of the culture supernatant for measurement of LDH activity. Results of the negative control (NC) were set to 1, and fold-change was calculated. Data represent arithmetic means ± SD of 4 (alveolar macrophages) or 3 (NRM2) independent experiments with 3 independent cultures per experiment, measured in triplicate. Statistically significantly different from NC: *p ≤ 0.05, **p ≤ 0.01, or ***p ≤ 0.001, Student’s *t*-test for unpaired values, two-tailed

To investigate induction of membrane damage, which can, in principle, be caused by both chemical and mechanical insults, LDH activity was measured in culture supernatants of AM (Fig. 3A) and NRM2 cells (Fig. 3B) treated for 24 h with RIF56008 or the two references materials. The technical positive control Triton^™^ X-100 in both cell-types induced a marked increase in LDH activity, amounting to fold-increases of 6.9 ± 2.99 (AM) and 26.5 ± 7.06 (NRM2 cells), relative to the negative control. In AM, slight, but significant increase was noted for amosite asbestos, with a maximal increase in LDH release of 1.8 ± 0.52-fold at the overload concentration of 50 µg/cm^2^ (*p* ≤ 0.001). No statistically significant induction of membrane damage was noticed in AM for both RIF56008 and the non-fiber material control RIF56008 ground (Fig. 3A).

In NRM2 cells, particularly at 50 µg/cm^2^, all materials statistically significantly induced membrane damage amounting to 2.5 ± 0.81-fold, 4.6 ± 1.32-fold and 7.8 ± 2.64-fold higher LDH activities for RIF56008 ground, RIF56008 and amosite asbestos treated cells (amosite asbestos > RIF56008 > RIF56008 ground), respectively, compared to the concurrent negative control (*p* ≤ 0.001; Fig. 3B). For both fiber samples, but not for RIF56008 ground, slight membrane damage was also induced at 5 µg/cm^2^ with 1.4 ± 0.31- (RIF56008) and 2.2 ± 0.33-fold (amosite asbestos) increases in LDH-activity, respectively.

To further evaluate the impact of overload and fiber-derived mechanical stress on membrane integrity, AM were incubated for 24 h with 5 or 50 µg/cm^2^ of the three materials. Subsequent sampling of supernatants was performed without or with movement/slight shaking of the cell culture plates to look for fiber morphology based, shaking-related piercing of the cell membrane potentially leading to artificial increase in LDH activity. Particularly at the highest amosite asbestos concentration (50 µg/cm^2^) no significant increase in LDH activity was observed without plate movement (Table 4), whereas movement of the plate induced a highly statistically significant increase in membrane damage. Cytotoxicity amounted to 18.9 ± 1.83%, compared to the technical positive control Triton^™^ X-100 set to 100% and the respective negative control (3.4 ± 1.25%). For RIF56008, movement of the cell culture plate at sampling led to 2.5-fold higher LDH activities than sampling without movement. This effect was absent in cultures treated for 24 h with the particle-like material control RIF56008 ground, therefore, indicating an artificial, fiber morphology-based effect.

**Table 4:** Influence of shaking during sampling for LDH release in rat alveolar macrophages after 24 h of incubation

Treatment	Concentration [µg/cm^2^]	Cytotoxicity [%] without shaking	Statistical significance	Cytotoxicity [%] with shaking	Statistical significance
Negative control	–	1.5 ± 0.41	–	3.4 ± 1.25	–
Triton X-100	1% (v/v)	100.0 ± 4.63	***	100.0 ± 2.99	***
RIF56008, ground	5	0.8 ± 0.17	n.s	3.0 ± 0.25	n.s
	50	3.2 ± 0.60	*	4.4 ± 1.01	n.s
RIF56008	5	0.3 ± 0.36	n.s	1.7 ± 0.62	n.s
	50	4.2 ± 0.70	**	10.3 ± 0.64	***
Amosite asbestos	5	0.0 ± 0.14	n.s	3.2 ± 0.49	n.s
	50	0.0 ± 1.29	n.s	18.9 ± 1.83	***

Arithmetic mean values of the respective technical positive controls were set to 100% cytotoxicity. Data represent arithmetic means ± SD of 3 biological replicates/cultures

*n.s *No statistically significant difference

Statistically significantly different from the respective negative controls: *p ≤ 0.05, **p ≤ 0.01, or ***p ≤ 0.001, respectively, Student’s *t*-test for unpaired values, two-tailed

#### Cell counts and cell proliferation

To investigate the influence of fiber treatment on cell counts, i.e., cell proliferation and/or cell death, AM and NRM2 cells (population doubling time approximately 13 h) were incubated for 24 h without or with 5 or 50 µg/cm^2^ of the different materials. At 50 µg/cm^2^ a highly significant (*p* ≤ 0.001) decrease in cell number was observed for all materials tested in both AM and NRM2 cells.

**Fig. 4:**
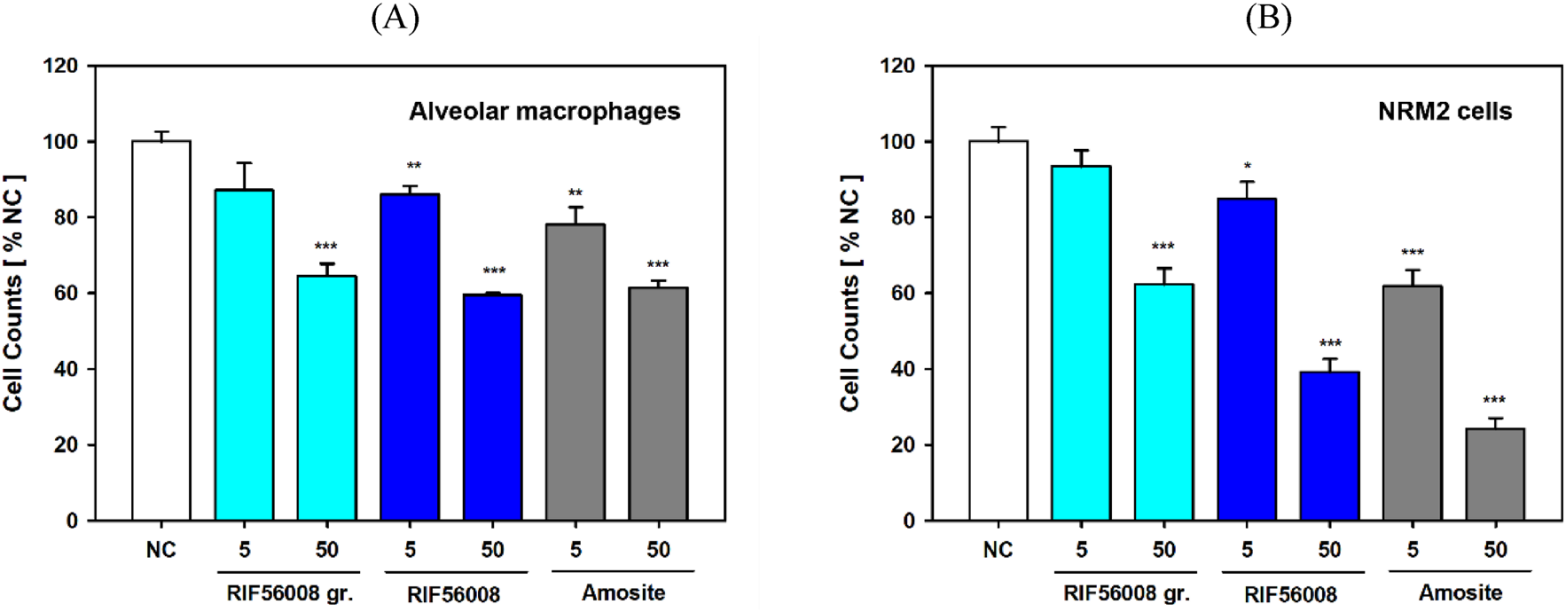
Reduction in cell counts in cultures of rat alveolar macrophages and NRM2 cells after 24 h of incubation with the particulate materials. AM (**A**) or NRM2 cells (**B**) were incubated for 24 h with the given concentrations of RIF56008 ground (RIF56008 gr.), RIF56008, or amosite asbestos (Amosite), before cell detachment and automatic cell counting were performed. Arithmetic means of the respective negative controls (NC) were set to 100%, and relative cell counts were calculated. Data represent arithmetic means ± SD of 3 biological replicates/ cultures. Statistically significantly different from NC: *p ≤ 0.05, **p ≤ 0.01, or ***p ≤ 0.001, Student’s *t*-test for unpaired values, two-tailed

In AM, RIF56008 ground, RIF56008, and amosite asbestos mediated nearly comparable reduction in cell counts to 64.5 ± 2.69, 59.4 ± 0.53, and 61.4 ± 1.57% of the negative control, respectively, whereas in NRM2 cells more marked effects and clear material differences were noted. Cell number decreased to 62.3 ± 4.24 (RIF 56008 ground), 39.2 ± 3.50 (RIF56008), and 24.2 ± 2.80% (amosite asbestos) of the negative control (Fig. 4). In both cell types decrease in cell count was concentration-dependent with lower effects at 5 than at 50 µg/cm^2^ and asbestos showing the strongest effect. At 5 µg/cm^2^, relative cell counts for asbestos-treated AM and NRM2 cells amounted to 78.1 ± 3.75 and 61.8 ± 4.29% of the respective negative controls, respectively (Fig. 4). For NRM2 cells clear ranking of materials was evident, i.e., amosite asbestos > RIF56008 > RIF56008 ground (Fig. 4B).

When investigating time-dependency in AM, comparable results were obtained for 4 and 24 h (Supplementary Tab. S2), but after 48 h the negative control cell count was reduced to 62.6 ± 10.79% of the 4 h negative control cell count, whereas both concentrations of RIF56008 ground and RIF56008 (5 and 50 µg/cm^2^) as well as amosite asbestos at 5 µg/cm^2^ showed almost comparable relative cell numbers around the negative control value. For amosite asbestos at 50 µg/cm^2^ cell count was further reduced to 30.74 ± 1.43% of the negative control after 4 h (Supplementary Table 1).

#### Induction of DNA strand breaks

To test for clastogenic activity, and thus for a genotoxic potential of the different materials, in vitro alkaline comet assays were performed in both AM and NRM2 cells after 24 h of incubation. Slide analysis and statistical data handling were predominantly done according to OECD 489 (“In Vivo Mammalian Alkaline Comet Assay”), as the currently effective guideline for in vivo comet assay studies.

**Fig. 5:**
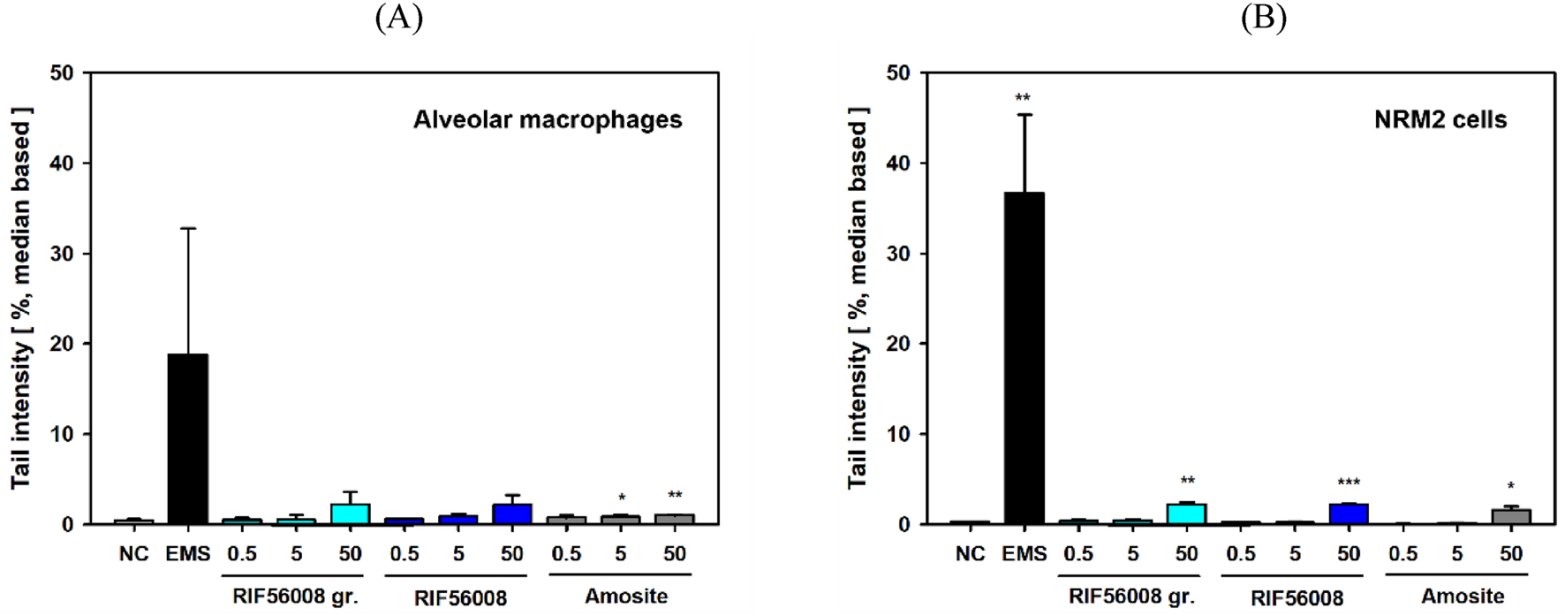
In-vitro alkaline comet assay with AM or NRM2 cells after 24 h of incubation with the particulate materials. AM (**A**) or NRM2 cells (**B**) were incubated for 24 h with the given concentrations of RIF56008 ground (RIF56008 gr.), RIF56008, or amosite asbestos (Amosite) before subjecting to the in vitro alkaline comet assay. Ethyl methanesulfonate (EMS; 1 µl/ml, 1 h) served as technical positive control and cell culture medium as technical negative control (NC). For each slide 100–150 cell nuclei were analyzed. Data represent arithmetic means ± SD of 3 independent experiments with three independent cultures per experiment. Arithmetic mean TI values are based on the arithmetic mean values of three independent experiments each derived from the three median values of the three biological replicates per experiment. Statistically significantly higher than the NC: *p ≤ 0.05, **p ≤ 0.01, or ***p ≤ 0.001, respectively, Welch’s *t*-test, one-tailed

In the in vitro alkaline comet assay with AM both RIF56008 ground (2.20 ± 1.390%) and RIF56008 (2.17 ± 1.071%) mediated a very slight and equal increase in arithmetic mean TI at 50 µg/cm^2^ only, as compared to the negative control (0.44 ± 0.157%; statistical significance not reached). No effect was seen for both 0.5 and 5 µg/cm^2^, as more in-vivo-relevant concentrations. Amosite asbestos mediated a biological as well as methodological irrelevant increase in TI at 5 and 50 µg/cm^2^ (0.85 ± 0.200% and 0.99 ± 0.082%, respectively), however with statistical significance, due to very low data variance. The methodological positive control EMS markedly increased arithmetic mean TI (18.81 ± 13.961%). Data for EMS, however, did not reach statistically significance, due to lower, but still marked increase in mean TI in one of the replicate experiments (Fig. 5A).

In the in vitro alkaline comet assay experiments with NRM2 cells, a comparable slight, but statistically significant increase in arithmetic mean TI was noted for all materials at the suggested overload concentration of 50 µg/cm^2^. Mean TI values amounted to 2.19 ± 0.274% (RIF56008 ground; *p* ≤ 0.01), 2.22 ± 0.135% (RIF56008; *p* ≤ 0.001), and 1.57 ± 0.447% (amosite asbestos; *p* ≤ 0.05), as compared to 0.28 ± 0.038% for the respective negative control (Fig. 5B). EMS mediated a marked and statistically significant increase in mean TI to 36.71 ± 8.641%. The two lower concentrations did not mediate any induction of DNA damage in NRM2 cells.

### Pro-inflammatory potential

To look for the pro-inflammatory potential of the three different materials in AM and NRM2 cells, cytokine-induced neutrophil chemoattractant 1 (CINC-1) was measured in culture supernatants after 24 h of incubation.

**Fig. 6:**
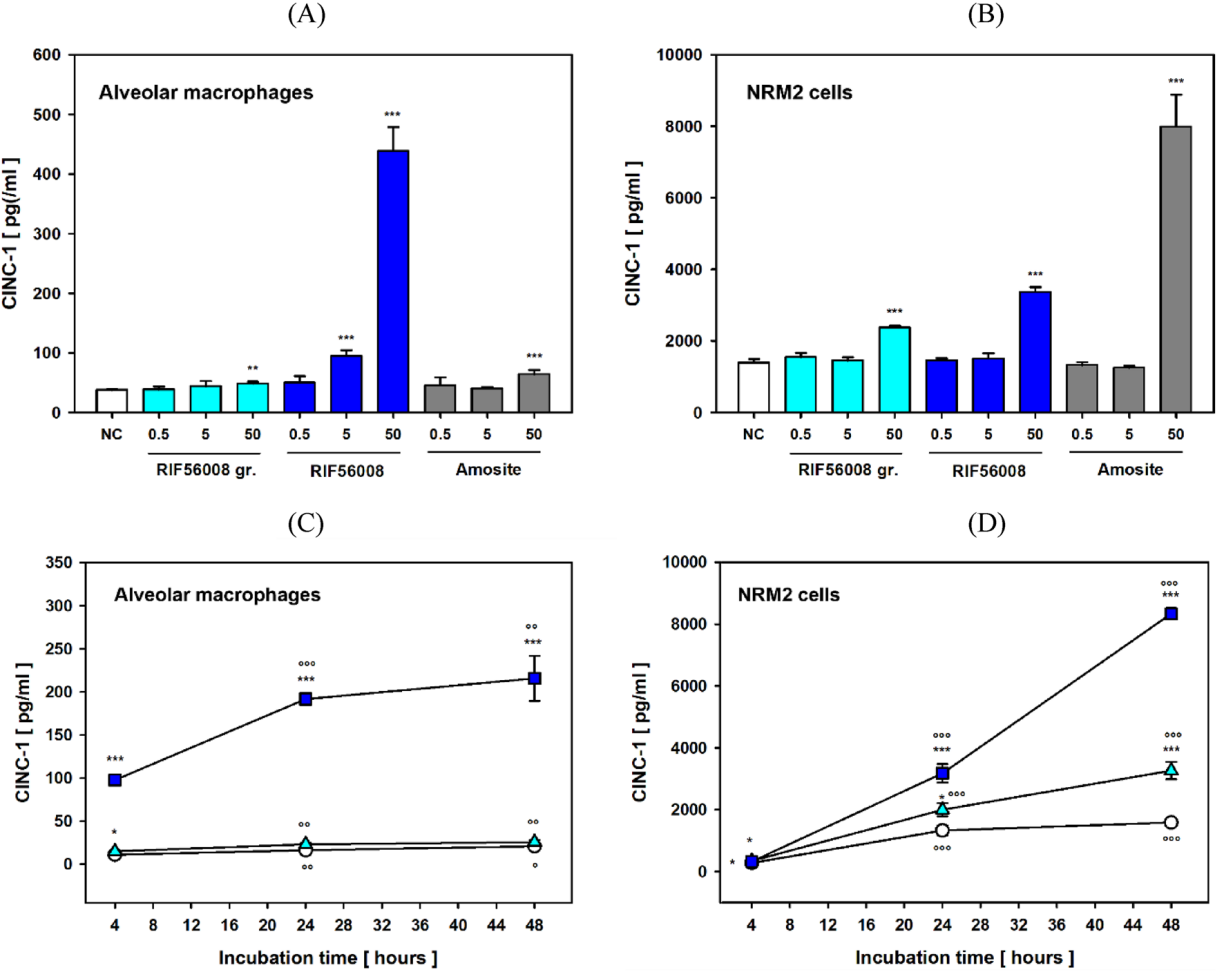
CINC-1 release in AM or NRM2 cells after 24 h of incubation with the different materials. Concentration-dependency, AM (**A**) or NRM2 cells (**B**) were incubated for 24 h with the given concentrations of RIF56008 ground (RIF56008 gr.), RIF56008, or amosite asbestos (Amosite), before sampling of culture supernatants for ELISA-based measurement of CINC-1. Data represent arithmetic means ± SD of 5 (AM) or 3 (NRM2 cells) independent biological replicates, each measured in duplicate. Statistically significantly different from the NC: **p ≤ 0.01 or ***p ≤ 0.001, Student’s *t*-test for unpaired values, two-tailed. Time-dependency, AM (**C**) or NRM2 cells (**D**) were incubated for 4, 24, or 48 h without (negative control, circles) or with 50 µg/cm^2^ of RIF56008 ground (triangles up) or RIF56008 (squares), before sampling of culture supernatants for ELISA-based measurement of CINC-1. Data represent arithmetic means ± SD of 3 independent biological replicates, each measured in duplicate. Statistically significantly different from the respective negative controls per timepoint: *p ≤ 0.05, **p ≤ 0.01, ***p ≤ 0.001; or from the respective 4 h timepoint: °*p* ≤ 0.05, °°*p* ≤ 0.01, °°°*p* ≤ 0.001; both Student’s *t*-test for unpaired values, two-tailed

In AM, RIF56008 highly significantly (*p* ≤ 0.001) and concentration-dependently induced CINC-1 release after 24 h of incubation at both 5 (94.3 ± 10.20 pg/ml) and 50 µg/cm^2^ (438.7 ± 39.88 pg/ml), compared to 37.6 ± 1.02 pg/ml for the concurrent negative control (Fig. 6A). Amosite asbestos, as non-biosoluble reference fiber also induced CINC-1 release at 50 µg/cm^2^, however, amounting to 63.7 ± 7.41 pg/ml only. For the particle-like material control RIF56008 ground, induction of CINC-1 release was very slight (47.9 ± 3.86 pg/ml), but nevertheless statistically significant at 50 µg/cm^2^.

Compared to AM, an about 37-fold higher basal CINC-1 release was noted in NRM2 cells after 24 h of incubation, amounting to 1389.9 ± 102.59 pg/ml (Fig. 6B). The lower concentrations of (0.5 and 5 µg/cm^2^) had no effect on CINC-1 release, whereas 50 µg/cm^2^ induced a highly statistically significant (*p* ≤ 0.001) increase in CINC-1 release for both RIF56008 ground (2373.0 ± 47.90 pg/ml), RIF56008 (3362.0 ± 137.81 pg/ml), and amosite asbestos (7983.3 ± 900.22 pg/ml), showing again the clear effect ranking of amosite asbestos > RIF56008 > RIF56008 ground.

When looking for time-dependency of RIF56008 ground- and RIF56008-mediated CINC-1 release in both cell types at 50 µg/cm^2^, increase in CINC-1 release was already seen in RIF56008-treated AM after 4 h, amounting to 97.5 ± 6.07 pg/ml, as compared to 10.9 ± 1.73 pg/ml for the respective negative control (Fig. 6C). However, no effect was noted in NRM2 cells after 4 h (Fig. 6D), indicating cellular uptake as one potential pre-requisite for a fiber-mediated pro-inflammatory effect. At 24 and 48 h of RIF56008 treatment, CINC-1 release in AM further increased in the absence of cytotoxicity (Fig. 3A), as compared to the respective negative controls. CINC-1 values for RIF56008 were maximal after 48 h of incubation with 215.7 ± 26.19 pg/ml, compared to 20.7 ± 3.94 pg/ml for the negative control (Fig. 6C). In NRM2 cells a time-dependent and statistically significant upregulation of CINC-1 release was evident for both the negative control, RIF56008 ground, and RIF56008, with RIF56008 demonstrating the steepest increase between 24 and 48 h of incubation (Fig. 6D). In contrast to AM, induction of CINC-1-release in NRM2 cells seemed to coincide with induction of membrane damage (Supplementary Fig. S3). After 2 days of exposure CINC-1 concentrations of 1585.2 ± 54.87, 3262.2 ± 283.00, and 8338.2 ± 182.07 pg/ml were evident for the negative control, RIF56008 ground, and RIF56008, respectively (Fig. 6D).

### Induction of binucleated NRM2 cells

In contrast to AM, NRM2 cells substantially proliferate, which might lead to disturbance of the cytoskeleton and the mitotic spindle by presence of fibers during cell division. It was previously demonstrated that in human mesothelial cells certain asbestos, glass and stone wool fibers can induce bi- and multinucleated cells (Pelin et al. 1995). Therefore, as additional endpoint, bi-/multinucleated NRM2 cells were analyzed after 48 h of treatment.

After 48 h of incubation, RIF56008 and amosite asbestos were shown to induce binucleated cells in a concentration-dependent manner, whereas RIF56008 ground mediated no effect (Table 5). RIF56008 treated NRM2 cells demonstrated 2-, 5-, and over tenfold, and NRM2 cells incubated with amosite asbestos 2-, 11-, and over 16-fold higher numbers of binucleated cells at 0.5, 5, and 50 µg/cm^2^, respectively, as compared to the negative control. At 50 µg/cm^2^ cell counts were significantly reduced to 19 (RIF56008) and 20% (amosite asbestos) of negative control (Table 5). As RIF56008 ground did not induce binucleated NRM2 cells, the effects seen for the other two materials represented most likely mechanical fiber effects.

**Table 5:** Binucleated cells per 2000 cells counted after treatment of NRM2 cells for 48 h without or with RIF56008 ground, RIF56008, or amosite asbestos. To estimate cytotoxicity relative cell counts were analyzed in parallel

Treatment	Concentration [µg/cm^2^]	Binucleated cells	Cell counts [% negative control]
Negative control	–	4	100
RIF56008, ground	0.5	2	108
	5	3	107
	50	5	77
RIF56008	0.5	8	104
	5	20	93
	50	39*	19
Amosite asbestos	0.5	8	108
	5	45	77
	50	65*	20

*Higher number of binucleated cells supposed, as analysis was limited by fiber material lying above the cells

## Discussion

The present study was conducted to advance in-vitro (geno)toxicity screening of MMVF. It aimed to technically evaluate potential cell models, biological endpoints, and methodological determinants and pitfalls. In vitro (geno)toxicity screening of industrially relevant MMVF in a controlled laboratory environment appears highly attractive, as complex, time-consuming, costly, and ethically questionable, animal experiments can be reduced. Using adequate cell models, relatively quick screening of many fiber types at different concentrations and incubation times seems possible, including gain of mechanistic insides. In addition, in vitro testing can be more easily customized and standardized than in vivo experiments and may guide MMVF development by safe-by-design strategies for development and use of safe and sustainable fiber materials in various industries. However, experimental design needs to be appropriate, reproducible, and predictive regarding the in vivo situation.

### Cell models

Besides the appropriate characterization of test materials, lung-relevant cell models with sufficient sensitivity and appropriate specificity/concordance are needed for meaningful (geno)toxicity screening of fibers. For human oriented hazard assessments, human cell models are clearly more relevant. However, new in vitro screening approaches should be confirmable in vivo. Since the rat is the standard species for in vivo biopersistence testing of fibers in compliance with Note Q of Regulation (EC) 1272/2008 (European Commission 1999) as well as lung fiber toxicity and carcinogenicity studies, rat cells seem better suited for initial efforts towards fiber-adapted in vitro screening approaches than human cells. Therefore, rat cells rather than human cells were chosen as cell models for this study, despite acknowledging possible differences in sensitivity compared to human cells. To avoid hypersensitivity, the best choice for in vitro screening of MMVF are primary cells or cell lines with functionally active p53 protein. This ensures appropriate responses to DNA damage, maintains genomic stability and normal chromosome counts as well as non-aberrant signal transduction pathways.

Since AM serve as the first line of defense in the respiratory tract, primary AM with intact p53 were chosen as first lung-relevant cell model in the present methodological in vitro investigation. The interaction of fibers with AM is the first prerequisite for both fiber clearance and adverse lung effects, such as release of cytokines and reactive oxygen species after fiber engulfment. AM represent a long-established, well-known, and sensitive cell model for in vitro fiber testing with reasonable correlation with parallel in vivo studies (Nguea et al. 2008; Ziemann et al. 2014, 2017; Creutzenberg et al. 2022). It was hypothesized that AM, as non-proliferating cell type should be less susceptible to morphology-driven adverse effects of fibers than proliferating cells, what could finally be demonstrated in the present study. Here fiber-induced membrane and DNA damage was nearly absent in rat AM after 24 h of incubation with non-overload concentrations of RIF56008. This could not be assigned to a lack in fiber engulfment, as fiber uptake was proven microscopically, and 24 h of incubation was thus long enough to ensure intracellular fiber exposure. Adhesion to fibers and subsequent engulfment of fibers by AM was previously shown to occur within 4 min of incubation (Miller et al. 1978; Luoto et al. 1994). Uptake of crocidolite fibers in Met5A human mesothelial cells was also previously demonstrated by flow cytometry. Fiber uptake into Met5A cells, however, increased with incubation time (number of cells with fibers was higher after 24 h than after 1 and 4 h of incubation) and dosage, but decreased with higher cell density (Yamashita et al. 2013). This suggests that part of RIF56008 fibers were engulfed without initiation of frustrated phagocytosis and subsequent membrane damage. In principle, this might be based on fiber chemistry and/or fiber dimensions shorter than the estimated AM mean diameter of 13.6 ± 0.4 µm (Krombach et al. 1997). Notably, Dörger et al. (2000) also did not observe marked induction of LDH release from rat AM after 24 h of incubation with 100 µg/ml of MMVF10 and MMVF21. In contrast, Shinji et al. (2005) demonstrated significant LDH release in Fischer 344 rat-derived alveolar macrophages after 18 h of incubation, but only at higher concentrations of 160 and 320 µg/ml of microglass fibers. No effect was observed at 40 and 80 µg/ml. It was finally concluded that the observed membrane damage resulted from disturbances of the cytoskeleton.

Primary rat mesothelial NRM2 cells were chosen as second lung-relevant cell model. These cells proliferate and were, therefore, suggested to be more prone to adverse effects of fibers, as fibers can physically disturb mitosis and cell division. This was indeed the case, when considering LDH release and reduction in cell counts. Mesothelial cells normally line serous cavities such as pleura and peritoneum. These cells produce serous fluids, and are involved in immune defense, wound healing, regulation of inflammatory processes, and transport and/or removal of foreign materials and infected cells (Mutsaers 2002). Notably, mesothelial cells are the primary targets for asbestos-mediated malignant mesothelioma, which originally prompted the development of non-carcinogenic asbestos alternatives such as MMVF. Irrespective of the fact that MMVF, and particularly biosoluble MMVF, seem unlikely to cause mesothelioma, when considering certain rat inhalation studies and epidemiological studies at workplaces (Bunn et al. 1993; De Vuyst et al. 1995; Hesterberg et al. 1995; Marsh et al. 2001), primary mesothelial cells represent an attractive and sensitive cell model for MMVF testing. Human primary mesothelial cells, rat pleural mesothelial cell, and the human MeT-5A mesothelial cell line, were both shown to internalize fibers and respond sensitively to fiber exposure (Pelin et al. 1995; Yegles et al. 1995; Cavallo et al. 2004; Yamashita et al. 2013). Notably, human MeT-5A cells, commonly used as a fiber-sensitive primary human mesothelial cell model, have been immortalized by transfection with the pRSV-T plasmid. These cells were also found to be more susceptible to the induction of bi- or multinucleated cells by MMVFs or milled MMVFs, compared to primary human mesothelial cells (Pelin et al. 1995). To avoid hypersensitivity, a primary mesothelial cell model with functional p53 protein such as NRM2 cells, seemed thus better suited. In the present study, non-immortalized NRM2 cells, nevertheless, demonstrated, as expected higher sensitivity towards adverse effects of RIF56008 and asbestos than AM. They clearly differentiated asbestos from MMVF samples in the rank order RIF56008 ground < RIF56008 < amosite asbestos, when considering membrane damage, CINC-1 release, reduction in cell counts, and induction of binucleated cells.

### Reference materials

Besides question-adapted cell models, defining appropriate negative and positive controls is crucial for setting up in vivo-relevant and predictive in vitro screening approaches. In the case of chemicals, negative controls may include solvent/vehicle controls, while positive controls could be chosen from the respective chemical space and/or represent endpoint-adapted technical positive controls such as Triton^™^ X-100 (for inducing membrane damage) or EMS (for inducing DNA damaging) used here. In contrast, testing of particles and fibers, should consider not only chemical characteristics, but also test item morphology, which per se can mediate adverse cell effects. Therefore, the use of particle- and fiber-like negative and positive controls is highly desirable, but not easy to realize. For short-term experiments with particles, non-soluble, chemically inert particles may be used as particle-like negative controls, like, e.g., aluminum oxide, and highly surface-reactive quartz DQ12 can serve as particle-like positive control in AM (Ziemann et al. 2014, 2017). However, choice of appropriate particle-like references requires consideration of both cell type and endpoints, as well as an understanding regarding mechanism of action.

The definition of suitable reference items becomes more complex for fibers with more than one morphological dimension. Fiber morphology per se can disturb membrane integrity, the cytoskeleton, and the mitotic spindle. Therefore, impact of fiber morphology was addressed in the present study using ground RIF56008 as a particle-like material control, which has the same chemical composition as the stone wool fiber RIF56008 used. The particle-like material control RIF56008 ground, e.g., clearly indicated that the slight induction of DNA strand breaks at 50 µg/cm^2^, in the absence of overwhelming cytotoxicity, was obviously independent of particle or fiber morphology and most likely represented a general, mechanical overload effect, with subsequent mechanical stress, as DNA strand break induction was in the same slight range for both RIF56008 and RIF56008 ground in both cell types. The approach to use ground fibers as material reference was previously used by Pelin et al. (1995), when testing asbestos and MMVF for the induction of bi- or multinucleated cells in different cell types, including primary human mesothelial cells and rat liver epithelial cells (no effect of particle-like material control) vs. transformed MeT-5A cells (significant effect of ground fiber material). In line with Pelin et al. (1995), in the present study, RIF56008 ground was nearly inactive or only slightly active in differentiated AM at non-overload conditions. However, it showed higher, but less marked adverse effects in proliferating NRM2 cells, compared to fibrous RIF56008. These results suggest that the effects observed were likely due to fiber morphology rather than chemical effects, but definition of fiber-like negative controls and using particle-like material controls can be highly challenging, if the experimental design involves a sensitive, dividing cell type, higher concentrations within the overload range, and endpoints sensitive to particulate materials. Even, when a fiber type shorter than the defined WHO fibers and without a highly reactive surface is used, unexpected morphology-dependent results may occur. For instance, in the present study, RIF56008, with a GML of the WHO fiber fraction lower than the estimated cell diameter (Krombach et al. 1997), strongly induced CINC-1 release in AM (Fig. 6A) in the absence of bacterial/endotoxin contamination, and not paralleled by marked membrane or DNA damage. However, presence of a considerable number of longer fibers cannot be excluded.

In contrast, the non-soluble reference fiber, amosite asbestos, with known carcinogenic potential, was almost inactive in rat AM (differentiated cell type), whereas it seems to represent an appropriate fiber-like positive control in NRM2 cells (proliferating cells). However, in AM after 48 h, but not 24 h, amosite asbestos mediated stronger reduction in cell counts at 50 µg/cm^2^ than RIF56008. This indicates that incubation times longer than 24 h might be needed in AM in vitro to detect adverse effects of amosite asbestos. But time-dependency of adverse asbestos effects seem to be endpoint specific, as Cullen et al. (1997) demonstrated production of tumor necrosis factor alpha in rat AM already after 24 h, with no effect of a stone wool fiber (MMVF 21). Additionally, Leinardi et al. (2023) recently demonstrated that both short (length < 5 µm) and long (length > 5 µm) amosite asbestos fibers were active in murine macrophages in vitro after 24 h of incubation, but they mediated different types of cell death, i.e., pyroptotic-related immunogenic cell death via Toll-like receptor 4 activation and particle sensing and asbestos mediated inflammation-triggered apoptosis via complex signaling pathway involving reactive oxygen species, respectively. This clearly reflects the dilemma of selecting appropriate fiber-like reference materials and points to cell type-, endpoint-, incubation time-, and concentration-dependency of fiber-mediated adverse effects. Such dependencies make comparisons of different studies using the same materials challenging, as also concluded by Yegles et al. (1995), when trying to correlate results of cytotoxicity and anaphase/telophase aberration analysis of 18 different fiber samples in rat pleural mesothelial cells with other in vitro studies using the same materials and with mesothelioma development in rats, after intrapleural inoculation of 10 of these fiber samples. Furthermore, Riganti et al. (2003) showed different adverse activity of short and long amosite asbestos fibers on A549 lung epithelial cells, with lower effects of short amosite asbestos fibers on redox metabolism, bringing, in addition, fiber length into play. Considering these examples in combination with the results of the present study, amosite asbestos cannot be in good conscience defined as a standard fiber-like positive control, based on too many variables, potentially altering in vitro response.

### Material concentrations and relevance of fiber characteristics

Justified, in vivo-relevant concentrations are key for establishing in vitro screening approaches to avoid misinterpretation and overestimation of adverse in vitro effects. However, as fiber effects in vivo are in part a consequence of long-term local fiber exposure, and it needs more than one cell type for fiber pathogenicity, fiber in vivo effects are hardly illustratable in vitro. In vitro screening, therefore, often needs higher fiber concentrations for induction of relevant adverse effects, but there is a fine line between meaningful in vitro concentrations and artificial overload effects, as demonstrated in the present study, after justified selection and use of 0.5 µg/cm^2^ (relevant for occupational exposure), 5 µg/cm^2^ (initial effects are expected to occur in vitro), and 50 µg/cm^2^ (considered an overload concentration). Here, adverse effects were mainly noted at the supposed overload concentration of 50 µg/cm^2^.

More than 30 years ago, overload was observed experimentally in vivo, in a study of pulmonary clearance of inhaled asbestos fibers in rats. Subsequently, Morrow (1988) developed a hypothesis based on the premise that physical overload of macrophages leads to a loss of mobility and impaired clearance of particulate or fibrous material. Already at that time, a threshold for the retained lung burden that will cause continuously increasing prolongation of particle lung clearance was given to be at a concentration that causes > 6% volume increase of alveolar macrophages, and impaired macrophage mobility was confirmed as a contributing factor to material overload in the lungs. This impairment also leads to additional effects, such as translocation of particles to the interstitium and pulmonary lymphatics, as well as persistent inflammatory response.

For in vitro studies, the overload situation is, however, very different and does not adequately reflect or mimics the complex mechanisms within the lung. Cellular overload generally involves overwhelming cellular responses and repair capacities, varying in concentration or particle/fiber number from one cell model to another. Most literature on particle overload in in-vitro testing systems focuses on nanomaterials. However, several differences have been identified in responses that complicate in vitro to in vivo extrapolation (IVIVE) approaches (Meldrum et al. 2022). The A549 lung epithelial cell line as well as the highly reactive macrophage cells (NR8383) have been investigated with various nanomaterials, and the onset of overload was defined. For TiO_2,_ overload occurred at concentrations of 100 and 200 µg/cm^2^ in A549 cells (Meldrum et al. 2022). For NR8383 cells, the overload threshold for several materials was identified at 6000 mm^2^/mL, based on the highest particle surface area–based concentration (Kroll et al. 2011; Wiemann et al. 2016, 2018). Thus, overloading an in vitro test system is an important consideration during testing, and the measures for determining thresholds need to be discussed. Overall, the methods for defining overload in vitro and in vivo are very different.

Besides the effects of material mass, fiber morphology per se seems to play a role, since the particle-like material control, RIF56008 ground, was always least active. Moreover, the experiment on the impact of plate movement showed artificially high LDH values, which were most likely due membrane piercing based on fiber morphology. But the present small data set is not comprehensive enough to accurately correlate test item characteristics with adverse effects in the two cell models used. GML, which is thought to be a key parameter (e.g., Yegles et al. 1995) could not be evaluated due to the limited testing of only two fiber types for induction of adverse effects. But some initial trends were observed in the present study. These include correlations between the number of WHO fibers and LDH release in both AM (50 µg/cm^2^) and NRM2 cells (at 5 µg/cm^2^), CINC-1 release in NRM2 cells (under supposed overload), and a reduction in cell counts in NRM2 cells at the in vivo relevant concentration of 5 µg/cm^2^. Additionally, the present dataset indicated correlations between GMD and LDH release and RICC in both cell types, and CINC-1 release in NRM2 cells. Here, specific surface seemed to be less important, but there was a tendency towards a correlation with the induction of binucleated cells at 5 µg/cm^2^, while particle number did not correlate with any observed endpoints. However, for meaningful judgments, further screening of additional fiber types with differences in GML, GMD, and specific surface are key. Notably, Yegles et al. (1995) demonstrated a correlation between the induction of aberrant anaphases/telophases and the number of “Stanton fibers”, which are defined as fibers with a length > 8 µm and a diameter of ≤ 0.25 µm. This integrates length, diameter, and fiber number, pointing to these fiber characteristics as potential key information in the context of in vitro fiber studies.

### CINC-1 release in primary rat alveolar macrophages

CINC-1 belongs to the CXC-chemokine family and possesses pro-inflammatory potential by, amongst others, promoting migration of neutrophils. Neutrophils can rapidly ingest and clear foreign bodies by phagocytosis with powerful H2O2 generation and intra- and extracellular burst, however, mechanistically different from AMs (Nordenfelt and Tapper 2011; Mayadas et al. 2014; Rosales 2018). CINC-1 was previously suggested a useful, and relatively early biomarker for the prediction of pulmonary toxicity of nanomaterials after both intratracheal instillation and inhalation exposure (Tomonaga et al. 2020). Unexpectedly, RIF56008 induced significant and concentration-dependent CINC-1 release in AM, mainly at overload, in the absence of bacterial/endotoxin contamination. Absence of a considerable effect in the RIF56008 ground-treated cells might indicate that fiber morphology represented an important determinant. In addition, it can be speculated that the production of ROS through frustrated phagocytosis or specific surface reactivity could have contributed, as ROS can trigger CINC-1 release (Handa et al. 2006). In this context, it should also be considered whether grinding of RIF56008 may have altered its potentially reactive surface characteristics, leading to surface passivation, and thus explaining lower activity of the RIF56008 ground fraction. For biosoluble particulate materials, a transient, acute inflammation is often observed in vivo in rat lungs, which resolves over time. Morimoto et al. (2016) demonstrated this for zinc oxide nanoparticles after both intratracheal instillation and inhalation, showing that initially high CINC-1 levels in BALF eventually resolved completely. The observed effect, however, appears to be multifactorial, with fiber morphology as only one determinant. It was particularly interesting that the amount of CINC-1 release and the rank order of RIF56008 and asbestos was clearly cell type-specific, when considering, in addition, CINC-1 release of the NRM2 rat mesothelial cells with statistically significant CINC-1 release after 24 h and more profound after 48 h at 50 µg/cm^2^ for all materials tested in the rank-order amosite asbestos >> RIF56008 > RIF56008 ground.

## Conclusions

The present data obtained from both non-proliferating cultured primary rat alveolar macrophages and proliferating primary rat NRM2 mesothelial cells using relatively easy-to-perform endpoints, clearly demonstrated that standardization of in vitro fiber (geno)toxicity screening still poses various challenges. Further mechanistic validation and comparison with preferably existing in vivo studies are necessary to approximate meaningful and in vivo relevant screening approaches. The present study, nevertheless, pointed to different key findings, may be, important for development of in vitro fiber screening concepts. For example, some endpoints, such as DNA strand break induction and LDH release, most likely revealed non-specific effects, based on fiber overload, with limited potential to distinguish between different fiber types, especially biosoluble fibers from non-dissoluble fibers like asbestos. In addition, short-term cell cultures (24 h of incubation), depending on the cell model used, might fail to capture effects related to fiber solubility and chemical composition. Additionally, fiber morphology and fiber number seem to represent the most important characteristics in short-term in vitro screening. To better integrate the parameters solubility and fiber chemistry into in vitro testing, longer incubation times or pre-incubation of fibers in artificial lung and/or subcellular fluids with subsequent incubation of cells with the resulting suspensions might be helpful.

The results of the present study, furthermore, pointed to marked cell type specificity concerning adverse effects, which can at least partly depend on the ability of the cell model to proliferate. Here, there was better differentiation of the two fiber types in proliferating, fiber-sensitive rat mesothelial cells, compared to AM. Therefore, predictivity of one cell type only for screening may not be sufficient, with subsequent under- or overestimation of adversity and potentially false-positive results. In this context, it must be carefully considered, if fiber screening approaches should be more predictive for the in vivo situation, or more sensitive to avoid false-negative results. Further discussion is also needed to determine whether a lung-relevant cell type or the capacity to differentiate fibers with high/low adverse potential and/or biosolubility/non-biosolubility is of higher priority, or do we need a combination of both, as approached in the present study.

The unexpected release of CINC-1, induced by RIF56008 in AM, also represents a hint towards screening of additional endpoints and mechanistic aspects, before definition of more standardizable screening approaches. For instance, specific mediator panels, which could also include eicosanoids like thromboxane, might offer greater predictivity than easy to measure endpoints like LDH release or cell counts. But this would also need further testing of a higher number of both biosoluble and non-biosoluble fiber samples. In this context it would be, e.g., crucial to compare RIF56008 with other MMVFs with low biosolubility and different morphologies, and to consider time dependencies in more detail by extending incubation times. Exploring disturbance of the cytoskeleton by immunocytochemistry might also represent a promising approach to screen for biological activity of mineral wools, since the cytoskeleton is involved in a lot of cellular processes, including phagocytosis and macrophage activation. As a future perspective, microarray technologies seem promising to identify specific differences in gene expression associated with various fiber types to derive from respective data meaningful/specific endpoints or endpoint panels.

In conclusion, to define an MMVF-adapted, predictive in vitro (geno)toxicity screening tool, it is crucial to carefully select reference chemicals/materials, endpoints, concentrations, and incubation times, based on in vivo relevance, as well as sensitivity and specificity of the chosen cell model(s). Additionally, further evaluation of endpoints is necessary, ideally with validation by preferably existing in vivo data regarding their predictivity.

## Supplementary Information

Below is the link to the electronic supplementary material.

Supplementary file1 (DOCX 3957 KB)

## Data Availability

The data that support the findings of this study are available from the corresponding author, upon reasonable request.
