# Supplementary material for: Methodological steps forward in toxicological in vitro screening of mineral wools in primary rat alveolar macrophages and normal rat mesothelial NRM2 cells
Source: Arch Toxicol. 2024 Sep 11;98(12):3949–71. doi: 10.1007/s00204-024-03855-7 (PMC11496320; doi:10.1007/s00204-024-03855-7)
Supplement: Supplementary file 1 — Supplementary file1 (DOCX 3957 KB) [file 204_2024_3855_MOESM1_ESM.docx]

**Supplementary Information:**

**Supplementary Table S1:**

**Table S1** Default values and MPPD 3.04 settings used to predict doses

|  | **Rat** | **Human** |
| --- | --- | --- |
| **Input Data - Airway morphology** | | |
| Model | Asymm. Multiple – Path | Yeh / Schum 5-Lobe |
| FRC (functional residual capacity) [ml] | 4.0 ml | 3300 ml |
| URT volume (Volume of upper airway tract) [ml] | 0.42 | 50.0 |
| **Input Data – Particle properties** | | |
| Density [g/cm^3^] | specific data | specific data |
| „Inhalability Adjustment“ | set | set |
| „MMAD“ | choose | choose |
| Diameter MMAD [μm] | specific data | specific data |
| GSD | specific data | specific data |
| **Input Data – Exposure Condition** | | |
|  | “Constant exposure“ | “Constant exposure“ |
| Aerosol concentration [mg/m³] | study NOAEC | study NOAEC |
| Breathing frequency [/min] | 102/min | 20/min for 8h (working day) |
| Tidal volume [ml] | 2.1 | 1040 |
| Inspiratory fraction | 0.5 | 0.5 |
| Pause fraction | 0 | 0.0 |
| Breathing scenario | nasal | oronasal-normal augmenter |
| **Input Data – Deposition / Clearance** | | |
|  | Deposition Only | Deposition Only |

Based on the results obtained with MPPD for the alveolar region further calculations were performed to obtain the deposited mass per cm^2^ alveolar surface. Therefore, the following assumptions were made:

- Aerosol concentration: 1.35 mg/m^3^
- Breathing frequency: 20/min
- Tidal volume: 1040 mL
- Exposure duration: 8 h/d
- 240 working days per year
- Alveolar surface: 627.000 cm^2^ (according to TRGS 910)
- Clearance not taken into account.

**Supplementary Fig. S1:**

| **Treatment** | **0 µg/cm^2^** | **0.5 µg/cm^2^** | **5 µg/cm^2^** | **50 µg/cm^2^** |
| --- | --- | --- | --- | --- |
| ***Primary rat alveolar macrophages*** | | | | |
| RIF56008 ground | **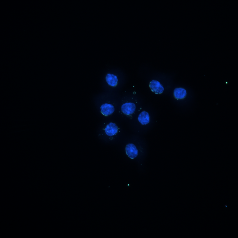** | 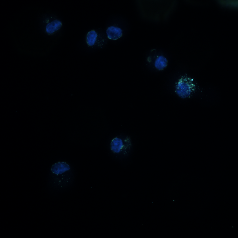 | 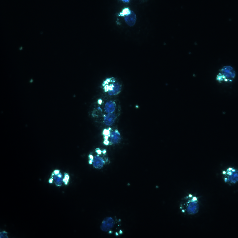 | 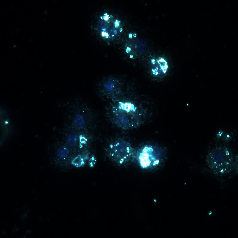 |
| RIF56008 | 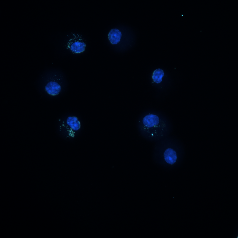 | 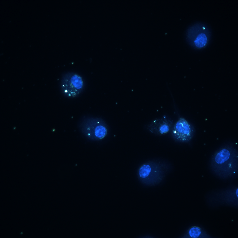 | 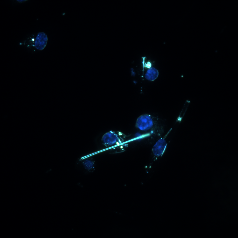 | 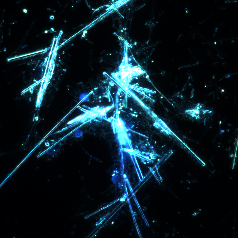 |
| Amosite asbestos | 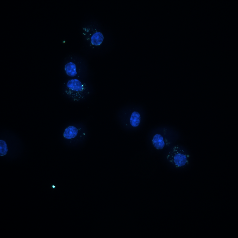 | 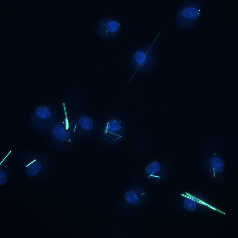 | 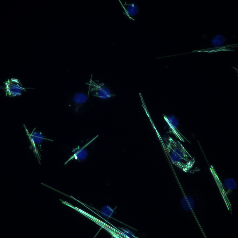 | 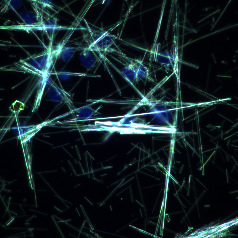 |
| ***NRM2 cells*** | | | | |
| RIF56008 ground | 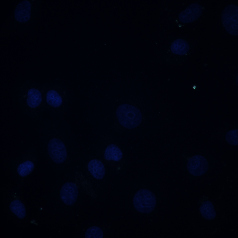 | 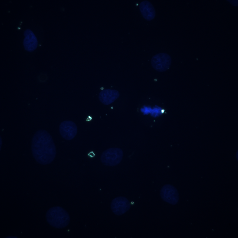 | 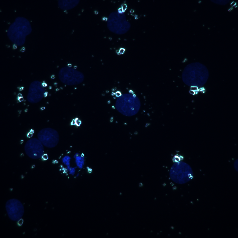 | 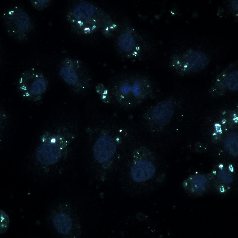 |
| RIF56008 | 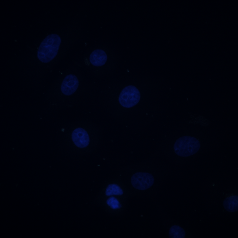 | 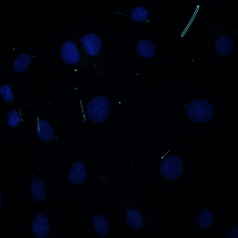 | 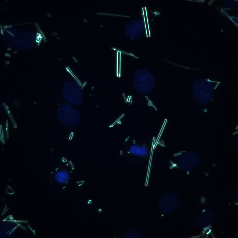 | 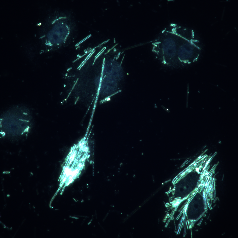 |
| Amosite asbestos | 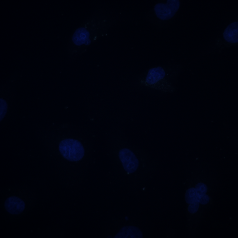 | 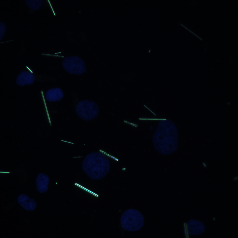 | 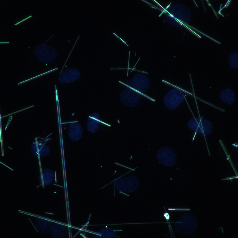 | 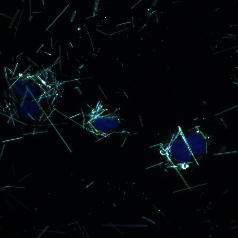 |

**Fig. S1** Representative fluorescence-coupled darkfield microscopy pictures from cellular uptake experiments with AM and NRM2 cells. AM or NRM2 cells were incubated for 24 h without (0 µg/cm^2^) or with the given concentrations (0.5, 5 or 50 µg/cm^2^) of RIF56008 ground, RIF56008 or amosite asbestos. Cells were subsequently fixed and cell nuclei stained with DAPI. Fluorescence-coupled darkfield microscopy was performed with a 100x oil objective and a final magnification of 1000x.

**Supplementary Fig. S2:**


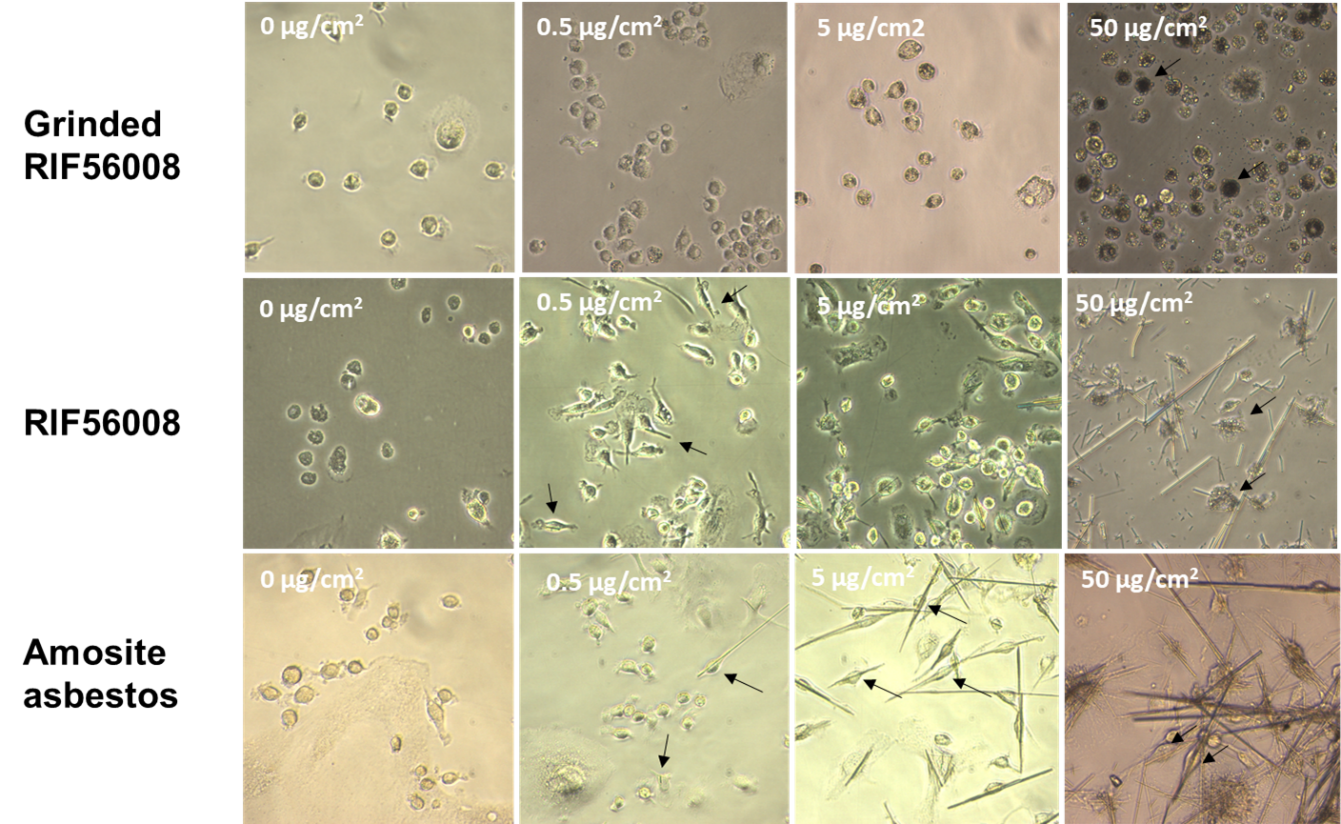


**Fig. S2** Representative light microscopic pictures showing primary rat alveolar macro­phages after 10 days of incubation with RF56008, RIF56008 ground, or amosite asbestos. Cells were incubated for 10 days without medium exchange in 24-well plates with the given concentrations of the three materials. At the end of treatment cells were directly inspected and documented by light microscopy.

**Supplementary Fig. S3:**


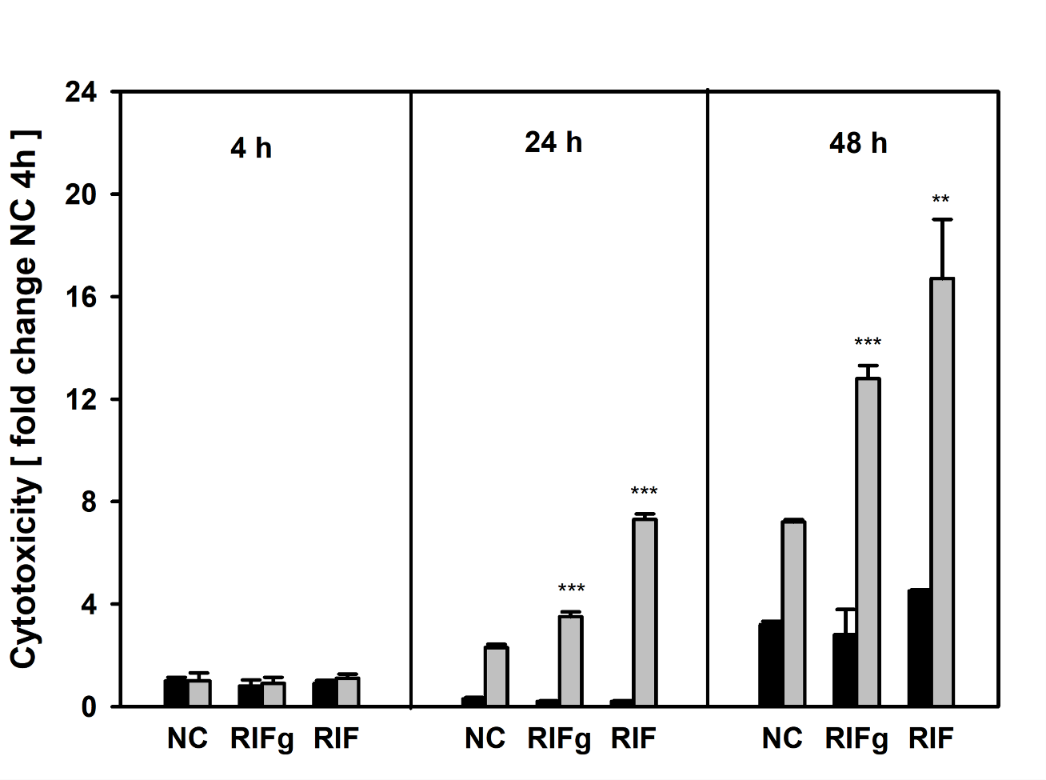


**Fig. S3** Time-dependent LDH release from AM or NRM2 cells after 4, 24 or 48 h of incubation without or with RIF56008 ground (**RIFg**) or RIF56008 (**RIF**). AM (**black bars**) or NRM2 cells (**grey bars**) were incubated for 4, 24, or 48 h without or 50 µg/cm^2^ the two materials, before sampling of the culture supernatant for measurement of LDH activity. Triton X-100 served as technical positive control and was set to 100% cytotoxicity. Subsequently, results of the respective 4 h negative control (**NC**) values were set to 1, and fold-change was calculated. Data represent arithmetic means ± SD of 3 independent biological replicates each measured in triplicate. Statistically significantly different from the respective NC values: ** *p* ≤ 0.01 or *** *p* ≤ 0.001, Student’s *t*-test for unpaired values, two-tailed.

**Supplementary Table S2:**

**Table S2** Time-dependent and concentration-dependent effects of RIF56008 ground, RIF56008, and amosite asbestos on cell counts in rat alveolar macrophage cultures.

| Treatment | Concentration | Cell counts [% negative control at 4 h] | | |
| --- | --- | --- | --- | --- |
|  | [µg/cm^2^] | 4 h | 24 h | 48 h |
| Negative control | - | 100.0 ± 4.07 | 120.3 ± 2.64 | 62.6 ± 10.79 |
| RIF56008 ground | 5 | 93.4 ± 3.78 | 104.8 ± 7.13 | 75.8 ± 2.71 |
|  | 50 | 74.1 ± 1.80 | 77.5 ± 3.24 | 56.6 ± 2.21 |
| RIF56008 | 5 | 84.1 ± 2.04 | 103.4 ± 2.23 | 67.8 ± 5.48 |
|  | 50 | 53.3 ± 0.61 | 71.48 ± 0.63 | 66.7 ± 0.93 |
| Amosite asbestos | 5 | 73.4 ± 0.92 | 93.9 ± 4.51 | 69.6 ± 5.98 |
|  | 50 | 62.7 ± 1.88 | 73.8 ± 1.88 | 30.7 ± 1.43 |
